# Development of Crispy, Antioxidant‐Rich Sultana Grape Chips via Hybrid Pre‐Drying and Explosion Puff Drying

**DOI:** 10.1111/1750-3841.71307

**Published:** 2026-07-23

**Authors:** Özgül Altay, Figen Kaymak‐Ertekin

**Affiliations:** ^1^ Faculty of Engineering, Department of Food Engineering Ege University Izmir Türkiye

**Keywords:** antioxidant activity, energy efficiency, explosion puff drying, hybrid drying, optimization, Sultana grape, texture

## Abstract

This study aimed to develop high‐quality, crispy, and nutritionally enriched chips from frozen Sultana grapes by optimizing pre‐drying strategies and explosion puff drying (EPD) parameters. Due to the challenges of drying high‐sugar fruits, pre‐drying methods, including hot air drying (HAD), microwave drying (MD), and hybrid combinations, were employed to reach specific moisture targets prior to EPD. A hybrid approach, consisting of MD at 360 W to 70% moisture followed by HAD at 60°C to 65%, proved most effective in preserving structural integrity, texture, and antioxidant potential. Subsequent optimization of EPD using a Box–Behnken design demonstrated that puffing temperature and time significantly affected expansion ratio (ER), crispness, adhesiveness, browning index (BI), and antioxidant activity (DPPH). The optimal conditions (104°C puffing temperature, 5 min puffing time, and 76°C system temperature) yielded grape chips with high expansion, enhanced crispness, reduced adhesiveness, and minimal browning (desirability: 0.814). Comprehensive analyses confirmed the superiority of the hybrid pre‐drying approach, resulting in lower water activity, improved color preservation (higher L* and lower BI), higher phenolic content and antioxidant activity, and enhanced energy efficiency (higher specific moisture extraction rate [SMER]) compared to single drying methods. Scanning electron microscopy (SEM) observations further revealed a more uniform and porous microstructure, supporting improved puffing behavior and textural quality. In conclusion, this study provides a scalable and energy‐efficient drying strategy for producing value‐added functional grape snacks and offers a practical framework for applying EPD technology to other high‐moisture fruits.

**Practical Applications:**

This study presents a scalable strategy for producing high‐quality, crispy grape chips from high‐moisture fruits using hybrid pre‐drying and explosion puff drying. The approach effectively improves texture, color, and antioxidant retention while enhancing energy efficiency. It can be readily integrated into existing industrial drying systems and applied to other sugar‐rich fruits. Additionally, it supports the valorization of surplus grapes, contributing to sustainable and value‐added food production.

## 1 Introduction

Grape (*Vitis vinifera* L.) is one of the most widely consumed fruits worldwide. Numerous studies have documented its diverse pharmacological properties, including antioxidant, cardioprotective, antiproliferative, anti‐inflammatory, antiaging, anti‐candida, antidiabetic, anti‐obesity, antimicrobial, and antispasmodic activities (Singh et al. 2023). Grapes are also rich in amino acids, B vitamins (B1 and B2), and essential minerals like potassium, magnesium, and iron. Their natural glucose content provides a quick source of energy, whereas bioflavonoids enhance vitamin C activity, serving as potent antioxidants that help prevent oxidative stress‐related damage, including the accumulation of low‐density lipoproteins (LDLs) in blood vessels. Additionally, grapes have been associated with improved digestion, hormonal regulation, immune system support, and ocular health protection (Khan et al. 2020).

In recent years, changes in consumer behavior driven by fast‐paced lifestyles, intensive work schedules, and evolving dietary patterns have fueled a growing demand for convenient, ready‐to‐eat (RTE) snack products. Such products, including chips, biscuits, nuts, and crackers, are valued for their portability, long shelf life, and ease of consumption. Although traditional snacks are often cereal‐based and high in fats and sugars, modern consumers increasingly seek options with added health benefits, prioritizing products that are nutritious, functional, and satisfying (Nor et al. 2013; Elmas et al. 2020). Within this trend, fruit‐based chips have gained particular popularity among health‐conscious individuals aiming to maintain balanced nutrition (Gao et al. 2022).

Drying is one of the most widely used techniques in RTE snack production, as it reduces water activity to levels (typically below 5%–10%) that inhibit microbial growth and enzymatic activity while helping retain flavor, aroma, and nutritional properties (Geankoplis 2006). A range of drying methods such as sun drying, hot air drying (HAD), microwave drying (MD), freeze drying, and explosion puff drying is selected based on the physicochemical characteristics of the food and the desired final product quality (Yi et al. 2016a). Among these, explosion puff drying (EPD) has emerged as a promising technology for producing high‐quality, crispy fruit snacks with enhanced sensory and nutritional attributes (Köprüalan et al. 2021). The EPD combines high pressure and temperature with vacuum to rapidly remove moisture, creating a porous, honeycomb‐like structure. Compared with conventional HAD, EPD generally results in improved crispness, greater expansion, and enhanced retention of sensory and nutritional properties owing to rapid moisture removal and the formation of a porous structure. In comparison with freeze drying, EPD may provide shorter processing times and lower operational costs while still yielding highly expanded snack products. Nevertheless, process optimization remains essential to achieve an appropriate balance between product quality and energy consumption. Previous studies have successfully applied EPD to a variety of food products, including pumpkin snacks (Köprüalan et al. 2021), mandarin snacks (Altay et al. 2023), white cheese snacks (Köprüalan et al. 2022), papaya snacks (Yi et al. 2016b), and pear snacks (Yi et al. 2016a). Foods processed using EPD often exhibit superior antioxidant activity, better retention of bioactive compounds, improved rehydration properties, and appealing texture and color (Du et al. 2013; Lyu et al. 2015; Bi et al. 2015). However, its direct application to high‐moisture products such as fresh grapes is limited, as insufficient internal pressure development results in poor puffing efficiency (Chen et al. 2017). To address this, pre‐drying treatments are commonly employed before EPD. Convective HAD is widely used in industry due to its simplicity and scalability (Hiranvarachat et al. 2011), but it is constrained by long processing times and low energy efficiency (Orikasa et al. 2014). MD, in contrast, provides rapid moisture removal by generating internal heating and vapor pressure that drives water toward the surface (Reyes et al. 2007; Swain et al. 2012). Nonetheless, MD can cause uneven drying, localized overheating, and structural damage due to nonuniform energy distribution (Roknul et al. 2014). Moreover, surface water removal must be efficient, which is better achieved with hot air because of its higher moisture‐carrying capacity compared to cold air. Consequently, hybrid drying methods that combine the rapid volumetric heating of MD with the efficient surface moisture removal of HAD have gained increasing attention for enhancing both energy efficiency and product quality (Song et al. 2017; Yi et al. 2016b). These approaches leverage the advantages of each method while mitigating their individual drawbacks, offering a synergistic solution for processing high‐moisture fruits such as grapes.

Despite the nutritional richness and functional potential of grapes, their drying poses challenges due to their high sugar content, large size, and waxy cuticle layer, all of which hinder moisture removal. Moreover, research on integrating hybrid‐drying techniques with explosion puff drying (EPD) for grape processing remains limited. There is a clear need to optimize pre‐drying parameters to enhance drying efficiency, improve expansion characteristics, and preserve the nutritional and sensory qualities of grape‐based snacks.

Although dried grape products are widely consumed, the development of crispy grape‐based snack products remains limited due to the high sugar content and sticky structure of grapes, which complicate drying and texture development processes. Conventional drying methods generally produce chewy or dense products with limited crispness and undesirable stickiness. In addition, increasing consumer demand for healthier snack alternatives has created a need for nutritious fruit‐based products with appealing sensory properties. Therefore, there is a growing need for alternative drying technologies capable of producing crispy, high‐quality grape snacks with improved structural and sensory properties. In this context, explosion puff drying (EPD) offers significant advantages because of its ability to generate porous structures, rapid moisture removal, and desirable crisp texture characteristics. This study aimed to produce grape chips as a healthy snack with high phenolic content and antioxidant activity, high crispness and expansion ratio (ER), and low stickiness and BI values. Frozen grapes were first dried under various conditions using HAD, MD, and hybrid HAD + MD methods to achieve different pre‐drying moisture contents. Subsequently, the grapes were dried to their final moisture content using EPD. Analytical results were collectively evaluated to determine the optimal pre‐drying moisture content, pre‐drying method, and drying conditions. The most suitable pre‐drying method and target moisture content were identified, after which the EPD process was optimized for grapes prepared under these conditions. The optimized parameters were then verified experimentally.

## 2 Materials and Methods

### 2.1 Materials

Sultana grapes (*V. vinifera* L. cv. Sultana), sourced fresh in season from the Manisa Viticulture Research Institute (Manisa, Türkiye), were stored at −24°C until the drying process. For each pre‐drying treatment, approximately 50 g of frozen grapes were used. To minimize variability, grape berries of similar size (14 mm diameter and 18 mm length) were carefully selected for each drying procedure. All drying experiments were conducted under the same sample loading conditions.

### 2.2 Methods

#### 2.2.1 Pre‐Drying Processes

**FIGURE 1:**
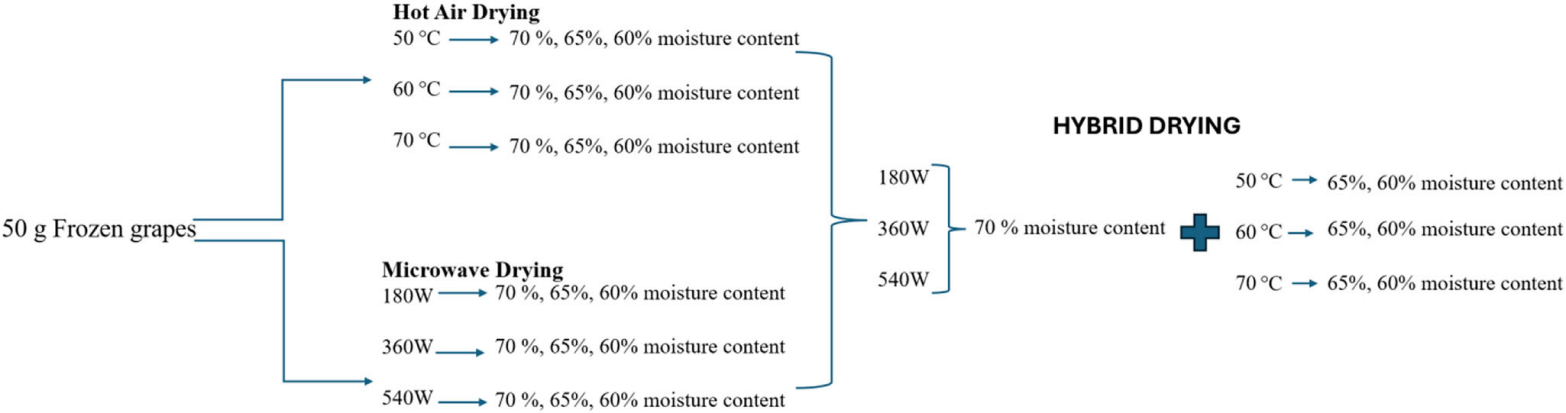
Schematic representation of the pre‐drying treatments applied before explosion puff drying (EPD). In hybrid drying treatments, grapes were first microwave dried to 70% moisture content and subsequently hot air dried to final moisture contents of 65% or 60%.

To determine the drying times required to achieve target moisture contents of 75%, 65%, and 60% (wet basis, wb) using HAD and MD, preliminary drying experiments were conducted using approximately 50 g of frozen grapes. Drying continued until a constant weight was attained. HAD was performed at an air velocity of 1.5 m/s and temperatures of 50°C, 60°C, and 70°C, whereas MD was carried out at microwave power levels of 180, 360, and 540 W. Different target moisture contents were selected to evaluate the effect of the pre‐drying degree on subsequent EPD performance and final product quality. Each drying condition was applied at all target moisture levels to systematically investigate the combined effects of drying intensity and residual moisture content on the characteristics of grape chips. On the basis of the preliminary experiments, the drying behavior was determined, and the drying durations required to reach each target moisture level were established. These drying times were subsequently used in the experimental treatments. Following the determination of drying times, the pre‐drying treatments (Figure 1) were applied as follows:

MD treatments: Frozen grapes were dried at 180, 360, and 540 W (MD 595, Arçelik, Türkiye) to moisture contents of 75%, 65%, and 60% (wb).

HAD treatments: Frozen grapes were dried at 50°C, 60°C, and 70°C (air speed: 1.5 m/s; Eksis Makine, Türkiye) to moisture contents of 75%, 65%, and 60% (wb).

Hybrid MD + HAD treatments: In the hybrid drying approach, MD was initially applied at power levels of 180, 360, or 540 W to rapidly reduce the moisture content of grapes to 70% (wb). Subsequently, HAD at 50°C, 60°C, or 70°C (air velocity: 1.5 m/s) was employed to further reduce the moisture content to final levels of 65% or 60% (wb). Hybrid drying treatments were applied only at these lower moisture levels, as they were considered more suitable for evaluating the combined effects of MD and HAD prior to EPD. This approach was designed to combine the rapid moisture removal capability of MD with the more controlled and uniform drying characteristics of HAD. Furthermore, the application of HAD during the later stage of drying was intended to minimize excessive thermal damage and uneven drying that may occur during prolonged microwave exposure.

#### 2.2.2 Explosion Puff Drying (EPD) Processes

The explosion puff dryer used in this study (Rapid Gıda Teknolojileri, Türkiye) was equipped with a compressor to increase environmental pressure, a vacuum pump to create a vacuum, infrared plate heaters as the heating medium, an infrared thermometer, an illuminator, and a Teflon/Polytetrafluoroethylene (PTFE)‐coated stainless steel sample tray. The system operates at a maximum absolute pressure of 190 kPa and a minimum absolute pressure of 25 kPa. During puffing, the maximum attainable material temperature is 150°C. The EPD process consists of two main stages: (i) the puffing stage and (ii) vacuum drying (Altay et al. 2023). Grapes pre‐dried to the target moisture content were further processed in the EPD unit under the following conditions: puffing pressure of 1.9 bar absolute (0.9 bar gauge), puffing temperature of 100°C, puffing time of 10 min, and system temperature of 60°C, yielding grape chips. The resulting puff‐dried grapes were analyzed for physicochemical properties (water activity, moisture content, color parameters [L*, a*, and b*], BI, and total color difference [Δ*E*]); structural properties (ER, bulk density, and texture [texture profile analysis, TPA: hardness and crispness]); bioactive properties (total phenolic content [TPC]); antioxidant activity (by 2,2‐Diphenyl‐1‐picrylhydrazyl [DPPH], ferric reducing antioxidant potential [FRAP], and2,2′‐azinobis‐(3‐ethylbenzothiazoline‐6‐sulfonic acid [ABTS]assays); sensory evaluation; and energy efficiency (specific moisture extraction rate [SMER expressed as kg water/kWh]).

Each drying experiment was performed in duplicate. The most suitable pre‐drying method and conditions were identified by evaluating ER, crispness, L* value, antioxidant activity (DPPH), overall sensory score, and SMER. Using grapes pre‐dried under these optimal conditions, the EPD process parameters were further optimized and validated.

#### 2.2.3 Optimization of Explosion Puff Drying Process for Pre‐Dried Grapes

To optimize the explosion puff drying (EPD) process, a Box–Behnken experimental design was applied using three independent variables: puffing temperature (*X*_1_: 90–110°C), puffing time (*X*_2_: 5–15 min), and system temperature (*X*_3_: 60–80°C). Five response variables were selected for evaluation: ER, crispness, adhesiveness, BI, and antioxidant activity (DPPH). A second‐order polynomial regression model was fitted to each response, and the statistical significance of model terms was assessed by analysis of variance (ANOVA). Multi‐response optimization was conducted using the desirability function approach, and experimental trials were performed to validate the model predictions. Following the explosion puff drying process, the products were analyzed for water activity, moisture content, color parameters (L*, a*, b*), BI, total color difference (Δ*E*), ER, bulk density, texture properties (TPA for hardness and crispness), TPC, antioxidant activity (DPPH, FRAP, and ABTS methods), sensory attributes, and statistical parameters. Among these, maximum ER and crispness, in‐range adhesiveness, high antioxidant activity (DPPH), and minimum BI were selected as the key responses for optimization.

A second‐order polynomial model (Equation 1) was fitted to describe the relationships between the independent variables and each selected response and evaluated using multiple linear regression analysis:

(1) $$\mathrm{Responses}=\beta_{0}+\sum_{i=1}^{k}\beta_{i}x_{i}+\sum_{i=1}^{k}\beta_{ii}x_{i}^{2}+\sum_{i=1}^{k-1}\sum_{j=i+1}^{k}\beta_{ij}x_{ji}\;\left(k=1,2,3\right)$$

where *X* is the independent variables, β_0_ is the constant coefficient, β *_i_* is the linear coefficient, β *_ii_* is the quadratic coefficient, β *_ij_* is the interaction coefficient, and *k* is the number of independent variables.

Significant model terms were identified through ANOVA. Optimization was performed by maximizing or minimizing the polynomial equations using both graphical (superimposition) and numerical (desirability function) approaches. The optimum operating conditions predicted by the model were experimentally validated in at least three independent trials.

### 2.3 Analyses

#### 2.3.1 Water Activity and Moisture Content

The moisture level of dried grape samples was determined using the gravimetric method, as stated by AOAC (1990). The sample's aw was measured using a water activity measurement instrument (Testo‐AG 400, Germany). Analyses were conducted in triplicate.

#### 2.3.2 Color Properties

L* (lightness), a* (redness), and b* (yellowness) values of the dried grape samples were measured using a Minolta CR‐400 colorimeter (Japan), and the results were expressed according to the *CIE Lab* system. The following equation was used to calculate Δ*E* of the samples, respectively (Köprüalan et al. 2021):

(2) $$\Delta E=\sqrt{\Delta L^{2}+\Delta a^{2}+\Delta b^{2}}$$

Five measurements were conducted for each analysis, and the average values were subsequently calculated.

#### 2.3.3 Expansion and Rehydration Ratio, Bulk Density

The ER was measured by comparing the volume of the same pre‐dried grape sample before and after the EPD process, and it was calculated according to the following equation (Chudy et al. 2019):

(3) $$\mathrm{ER}=\frac{V2-V1}{V1}\times 100$$

Rehydration ratio (RR) was calculated gravimetrically as the ratio of the rehydrated sample mass to the initial dried sample mass. The bulk density value of grapes dried by the EPD method was calculated using the ratio of sample weight to volume (kg/m^3^) (Altay 2020).

#### 2.3.4 Texture Analysis

The texture of the samples was measured using a Texture Analyzer (TA‐XT2, Stable Micro Systems, Haslemere, UK) equipped with a flat cylindrical probe (P/36R) and operated in TPA mode. Hardness was defined as the maximum compression force (N) required fracturing the sample, whereas crispness was quantified as the number of force peaks detected during compression (Yi et al. 2016a). The pretest, test, and posttest speeds were all set to 2.0 mm/s, with a test distance of 5 mm and a trigger force of 5.0 g. A 30 kg (294.2 N) load cell was used for all measurements. Ten replicates were performed per treatment, and average values were calculated.

#### 2.3.5 TPC and Antioxidant Activity

The TPC of the samples was determined by modifying the Folin–Ciocalteu method (Escarpa and González 2001), and the TPC of the samples was expressed as milligrams of gallic acid equivalent (GAE)/100 g of dry matter (DM). Antioxidant activity analysis was performed using DPPH, FRAP, and ABTS methods.

DPPH assay (2,2‐diphenyl‐1‐picrylhydrazyl radical scavenging capacity) analyses were analyzed spectrophotometrically at 517 nm using a Shimadzu UV‐1800 spectrophotometer. The total antioxidant activity of the samples was calculated using the equation derived from the standard calibration curve created with the Trolox standard and was expressed as milligrams of Trolox equivalent antioxidant capacity (TEAC)/100 g of DM (Akçay‐Salık et al. 2025).

FRAP assay analyses were performed spectrophotometrically at 593 nm using a Shimadzu UV‐1800 spectrophotometer (F. L. Song et al. 2010). The total antioxidant activity of the samples was calculated using the equation derived from the standard calibration curve created with the ascorbic acid standard and was expressed as milligrams of ascorbic acid/100 g of DM.

ABTS assay: The antioxidant activity of dried grapes was determined following the method of Ré (1998). Briefly, to prepare the ABTS radical cation (ABTS^+^) solution, 10 mg of 2,2′‐azino‐bis (3‐ethylbenzothiazoline‐6‐sulfonic acid) (ABTS, Sigma‐Aldrich) was dissolved in 2.57 mL of distilled water, and 37.5 mg of potassium persulfate (Sigma‐Aldrich) was dissolved in 1 mL of distilled water. These solutions were mixed and stored in the dark at room temperature for 12–16 h. Subsequently, 1 mL of the ABTS·^+^ solution was diluted with ethanol to achieve an absorbance of 0.70 ± 0.02 at 734 nm (initial absorbance value). A mixture of the prepared ABTS·^+^ solution and the dried grape extract was incubated in the dark for 6 min, after which its absorbance was measured at 734 nm using a spectrophotometer (Agilent Technologies, Cary 60 UV–Visible). The percentage inhibition of ABTS^+^ was calculated using the following equation:

(4) $$\mathrm{ABT}S^{+}\left(\%\right)=\left(A_{i}-A_{F}\right)\times 100/A_{i}$$

where *A_i_* is initial absorbance value, and *A_F_* is final absorbance value.

#### 2.3.6 Sensory Evaluation

Ten semi‐trained panelists evaluated the sensory properties of dried grapes produced using MD + EPD, HAD + EPD, and MD + HAD + EPD drying methods. Sensory attributes, including color, appearance, adhesiveness, crispness, and overall acceptability, were assessed using a nine‐point hedonic scale (Altay et al. 2022). Samples were served at room temperature in a randomized order and identified using three‐digit random codes. Water was provided to the panelists for palate cleansing between sample evaluations. In addition, the panelists rated their overall impression of each sample. The panelists, who were specifically selected for sensory evaluation, received product‐specific training conducted by a team leader experienced in dried fruit and vegetable products. The training familiarized the panelists with the sensory evaluation scale, attribute definitions, and the expected product characteristics associated with the different drying processes.

#### 2.3.7 Specific Moisture Extraction Rate (SMER)

To determine energy consumption, an electricity meter (Köhler, AEL. TF.04, Türkiye) was connected to the device, and electricity consumption was measured during the drying process. SMER values were calculated using the formula provided in the following equation (Baysan et al. 2022):

(5) $$\mathrm{SMER}=\frac{\mathrm{Mass}\;\mathrm{of}\;\mathrm{water}\;\mathrm{removed}\;\mathrm{from}\;\mathrm{the}\;\mathrm{product}}{\mathrm{Total}\;\mathrm{energy}\;\mathrm{consumption}}$$

#### 2.3.8 Particle Morphology

The particle morphology of dried grapes was examined using a scanning electron microscope (SEM) (FEI Quanta250 FEG, SEM). Samples were mounted on aluminum stubs with double‐sided tape and sputter‐coated with a thin layer of gold. Coated samples prepared under optimum conditions were observed at an acceleration voltage of 5 kV. Images capturing both the internal and external structures of the samples were obtained and evaluated in conjunction with the SEM analysis.

#### 2.3.9 Statistical Analysis

The most suitable pre‐drying method for grape samples was determined by evaluating the effects of HAD temperature, microwave power, and pre‐drying moisture content on the quality characteristics of the puff‐dried products. The effects of these variables were analyzed using ANOVA with Statistical Package for the Social Sciences (SPSS) software (IBM SPSS Statistics Base 22.0). Significant differences among mean values were determined using Duncan's multiple range test at a significance level of *p* < 0.05. All experiments were conducted in duplicate.

## 3 Results and Discussion

### 3.1 Nutritional (TPC and Antioxidant Capacity) Properties of Pre‐Dried Grapes

**FIGURE 2:**
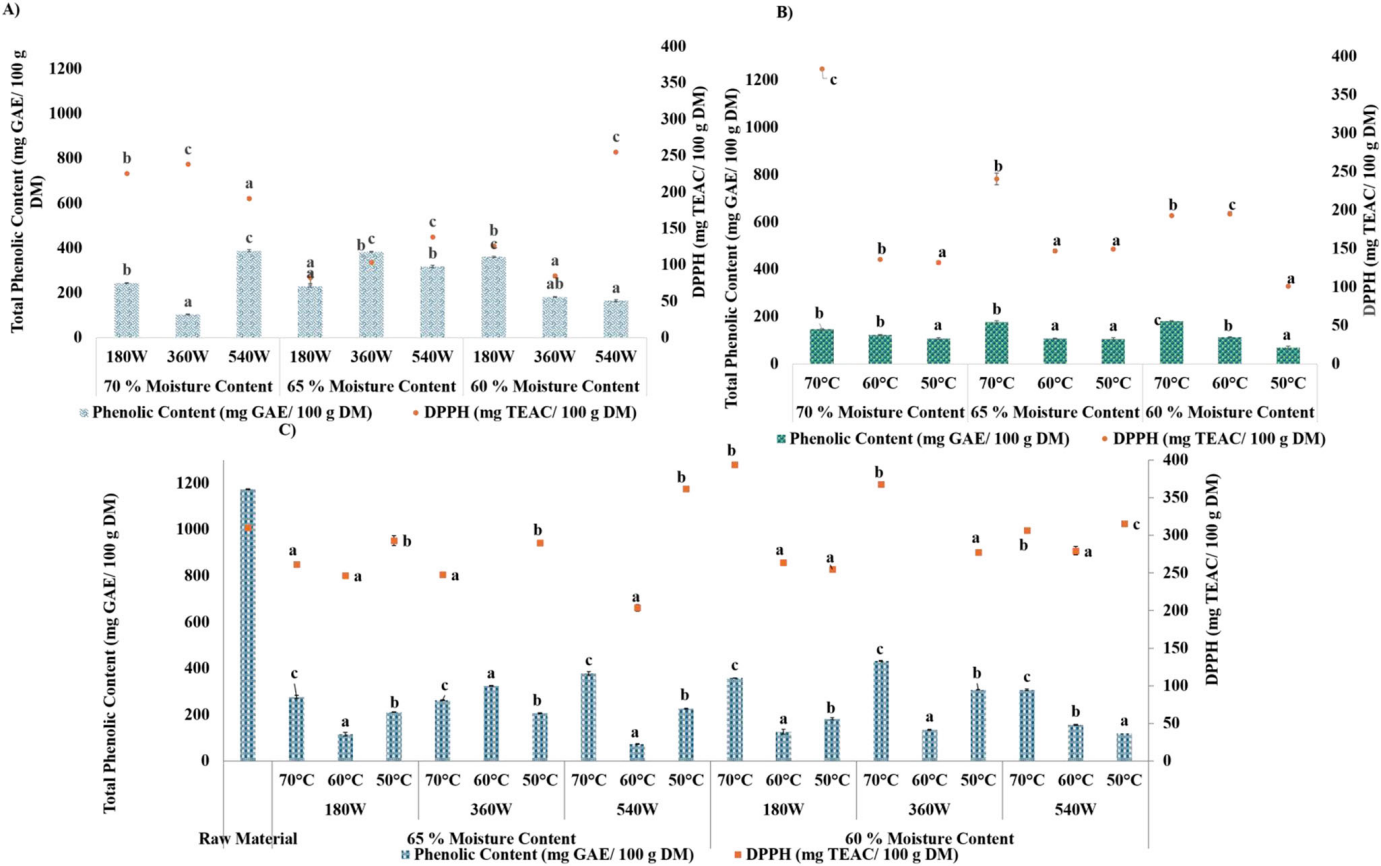
Antioxidant activity (DPPH) and TPC of grapes dried with (A) MD, (B) HAD, and (C) hybrid pre‐drying methods and then treated with EPD. Different superscript letters indicate significant differences (*p* < 0.05) among drying treatments within the same moisture content. DM, dry matter; GAE, gallic acid equivalent; TEAC, Trolox equivalent antioxidant capacity.

Figure 2 presents the antioxidant activity (DPPH) and TPC of grapes subjected to MD, HAD, and hybrid pre‐drying methods prior to EPD treatment.

Both the pre‐drying method and the drying conditions had a pronounced impact on these critical quality attributes, as evidenced by the significant variations among treatments. For antioxidant activity (DPPH), MD‐treated samples consistently exhibited higher values than those obtained with HAD. This finding is consistent with previous reports indicating that the rapid heating mechanism of MD can minimize thermal degradation of heat‐sensitive bioactive compounds such as polyphenols and flavonoids (Chandrasekaran et al. 2013; Zielinska et al. 2018). However, the effect of microwave power on antioxidant retention may vary depending on drying conditions and moisture content, as microwave heating can simultaneously shorten drying time while increasing the risk of localized overheating. In the present study, higher microwave power levels generally resulted in greater retention of TPC and DPPH values, which may be attributed to shorter drying durations and reduced overall thermal exposure. In contrast, HAD‐treated samples showed comparatively lower antioxidant activity and TPC values. The combined MD + HAD approach yielded superior results, effectively balancing the rapid moisture removal of MD with the uniform heat transfer of HAD. Bhattacharjee et al. (2024) reported that hybrid drying can improve drying uniformity and enhance the retention of heat‐sensitive compounds by utilizing the complementary advantages of the two methods. Altay et al. (2023) investigated the effects of different pre‐drying methods, including freeze drying, osmotic drying, MD, and HAD, on the production of puff‐dried mandarin chips. The study concluded that the combination of HAD and MD as a pre‐treatment, followed by EPD, represents an effective and alternative approach for transforming mandarins into a healthy snack while preserving their nutritional and textural properties. For TPC, MD‐pretreated grapes retained the highest phenolic levels, followed by MD + HAD and then HAD. As phenolic compounds, key contributors to antioxidant activity and health‐promoting effects are highly sensitive to prolonged heat exposure, the shorter drying times and moderated thermal load in MD + HAD likely contributed to better retention than in HAD alone.

Table 1 presents the antioxidant activity results assessed by FRAP and ABTS assays under the different drying conditions, whereas Table 2 provides statistical analyses confirming the significance of these differences. In the raw material, the FRAP value was 1110 mg AA/100 g DM, and the ABTS value was 99%.

**TABLE 1:** Antioxidant capacity of pre‐dried grapes (ferric reducing antioxidant potential [FRAP] and ABTS assays) after explosion puff drying under different microwave, hot air, and hybrid drying conditions.

	*Microwave drying*		
Moisture content (%)	Microwave power (W)	FRAP (mg AA/100 g DM)	ABTS (%)
70	180	193.08^c^ ± 1.14	97.96^c^ ± 0.24
	360	95.01^a^ ± 1.02	89.46^b^ ± 2.35
	540	99.66^b^ ± 6.49	80.92^a^ ± 1.52
65	180	178.23^a^ ± 3.09	97.07^a^ ± 1.30
	360	280.75^b^ ± 6.78	96.10^ab^ ± 1.38
	540	320.11^c^ ± 3.50	98.21^b^ ± 0.18
60	180	263.92^c^ ± 2.80	97.48^a^ ± 0.99
	360	248.81^b^ ± 3.98	98.46^a^ ± 0.10
	540	144.74^a^ ± 6.77	97.80^a^ ± 0.22

*Hot air drying*			
	Air temperature (°C)	**FRAP (mg AA/100 g DM)**	**ABTS (%)**
70	70	149.07c ± 2.86	90.45^b^ ± 0.81
	60	121.78b ± 3.17	81.52^a^ ± 2.56
	50	111.67^a^ ± 2.50	72.33^a^ ± 12.90
65	70	111.67^a^ ± 1.03	83.72^b^ ± 3.38
	60	90.77^c^ ± 3.26	66.19^a^ ± 0.90
	50	119.07^b^ ± 4.92	62.22^a^ ± 4.19
60	70	251.88^c^ ± 0.44	86.40^c^ ± 0.00
	60	186.86^b^ ± 4.08	54.31^b^ ± 0.23
	50	73.096^a^ ± 6.43	50.16^a^ ± 1.37

*Hybrid drying*				
	**Microwave power (**W)	**Air temperature (**°C)	**FRAP (mg AA/100 g DM)**	**ABTS (%)**
65	180	70	415.46^c^ ± 4.21	87.90^a^ ± 0.15
		60	286.91^b^ ± 3.57	98.35^b^ ± 0.06
		50	190.40^a^ ± 1.52	87.21^a^ ± 1.17
	360	70	259.49^b^ ± 3.78	92.98^b^ ± 0.14
		60	295.08^c^ ± 0.07	84.99^a^ ± 4.30
		50	220.96^a^ ± 3.78	98.13^c^ ± 0.32
	540	70	240.11^b^ ± 1.68	98.96^c^ ± 0.13
		60	288.05^c^ ± 3.36	94.69^a^ ± 0.69
		50	228.35^a^ ± 2.37	97.49^b^ ± 0.67
60	180	70	204.27^c^ ± 4.21	97.74^a^ ± 0.09
		60	145.33^b^ ± 3.57	98.00^b^ ± 0.23
		50	227.32^a^ ± 1.52	98.06^b^ ± 0.00
	360	70	203.01^b^ ± 3.78	95.73^a^ ± 4.11
		60	104.48^a^ ± 0.07	97.72^a^ ± 0.36
		50	242.22^c^ ± 3.78	97.30^a^ ± 0.81
	540	70	116.27^a^ ± 1.68	98.63^b^ ± 0.18
		60	158.57^b^ ± 3.36	93.79^a^ ± 1.96
		50	243.86^c^ ± 2.37	98.24^b^ ± 0.09

*Note*: Different letters in the same column are significant at the *p* < 0.05 level.

Abbreviation: DM, dry matter.

**TABLE 2:** Statistical analysis results for antioxidant activity (DPPH, ferric reducing antioxidant potential [FRAP], and ABTS) and total phenolic content (TPC) across different pre‐drying methods and conditions.

*p* value					
Source	SD	DPPH (mg TEAC/100 g DM)	FRAP (mg AA/100 g DM)	ABTS (%)	TPC (mg GAE/100 g DM)
**Microwave drying**					
Modal	8	0.000*	0.000*	0.000*	0.000*
Intercept	1	0.000*	0.000*	0.000*	0.000*
*A*	2	0.000*	0.000*	0.000*	0.000*
*C*	2	0.000*	0.000*	0.000*	0.000*
*A* × *C*	4	0.000*	0.000*	0.000*	0.000*
*R* ^2^		0.999	0.999	0.988	0.977
**Hot air drying**					
Modal	8	0.000*	0.000*	0.000*	0.000*
Intercept	1	0.000*	0.000*	0.000*	0.000*
*B*	2	0.000*	0.000*	0.000*	0.000*
*C*	2	0.000*	0.000*	0.000*	0.000*
*B* × *C*	4	0.000*	0.000*	0.000*	0.000*
*R* ^2^		0.999	0.999	0.946	0.994
**Hybrid drying**					
Modal	17	0.000*	0.000*	0.000*	0.000*
Intercept	1	0.000*	0.000*	0.000*	0.000*
*A*	2	0.000*	0.000*	0.000*	0.000*
*B*	2	0.000*	0.000*	0.000*	0.000*
*C*	1	0.000*	0.000*	0.000*	0.000*
*A* × *B*	4	0.000*	0.000*	0.000*	0.000*
*B* × *C*	2	0.000*	0.000*	0.000*	0.000*
*A* × *C*	2	0.000*	0.016*	0.000*	0.000*
*A* × *B* × *C*	4	0.000*	0.000*	0.000*	0.000*
*R* ^2^		1.000	1.000	0.963	0.998

*Note*: A: Microwave power (W), B: air temperature (°C), C: predicted moisture (%).

Abbreviations: DM, dry matter; GAE, gallic acid equivalent; TEAC, Trolox equivalent antioxidant capacity.

*Significant at *p* < 0.05.

Antioxidant activity results presented in Tables 1 and 2 provide a detailed comparison of MD, HAD, and MD + HAD in terms of their effects on FRAP and ABTS values. Statistical analysis (Table 2) confirmed that microwave power, air temperature, and moisture content significantly influenced antioxidant retention (*p* < 0.05), emphasizing the need to optimize drying parameters to preserve bioactive compounds. For MD, the effect of microwave power on antioxidant activity depended on the moisture content of the samples. The highest antioxidant activity was achieved at 70% moisture content and lower microwave power (180 W), whereas higher power levels (360 and 540 W) caused declines in FRAP and ABTS values. Similarly, Zielinska et al. (2018) investigated the effectiveness of microwave pre‐treatment at 100, 500, and 800 W on the drying kinetics of whole cranberries. The study concluded that the lowest microwave power (100 W) resulted in the highest retention of phenolic compounds, greater antioxidant activity, and an appealing color. This reduction can be explained to localize overheating at high intensities, which accelerates the degradation of heat‐sensitive phenolic compounds (Brown et al. 2012). At 65% and 60% moisture content, however, increasing microwave power improved FRAP values (e.g., 320.11 mg AA/100 g DM at 540 W, 65% moisture), suggesting that residual moisture may act as a protective barrier against oxidative degradation. The strong correlations between drying parameters and antioxidant retention were supported by high *R* ^2^ values (0.999 for FRAP, 0.988 for ABTS, and 0.977 for TPC). HAD generally resulted in greater antioxidant losses compared to MD and MD + HAD, particularly at lower drying temperatures (50–60°C), where prolonged exposure likely promoted oxidative and enzymatic degradation of phenolic compounds (Roknul et al. 2014). However, at 70°C and 60% moisture content, FRAP peaked at 251.88 mg AA/100 g DM, indicating that higher temperatures can help preserve antioxidants by shortening drying time and reducing enzymatic activity. ABTS values remained consistently lower in HAD, likely due to slower heat transfer and extended processing times, which facilitate polyphenol oxidation. Statistical analysis also showed that air temperature and its interaction with moisture content (BC) significantly affected all antioxidant parameters (*p* < 0.05), confirming the detrimental effect of prolonged heat exposure. The MD + HAD method proved most effective for antioxidant retention, yielding the highest FRAP values (415.46 mg AA/100 g DM at 180 W and 70°C, 65% moisture). This suggests that combining microwave and HAD offers synergistic benefits, rapid moisture removal from MD followed by uniform heat transfer from HAD, minimizing thermal degradation. Interestingly, at 60°C and 50°C, higher microwave power (360 and 540 W) enhanced antioxidant retention, indicating that moderate temperatures coupled with controlled microwave exposure can limit oxidative damage. Similarly, Maskan (2000) and Bhattacharjee et al. (2024), as a result of their work, reported that hybrid drying combines the advantages of MD's rapid moisture diffusion and HAD's uniform heat transfer, thereby reducing total drying time and preserving bioactive compounds. The three‐way interaction between microwave power, air temperature, and moisture content (ABC) was highly significant (*p* < 0.05) for all antioxidant parameters, underscoring the importance of multifactor optimization in hybrid drying. Optimization is particularly relevant for grapes, where the waxy cuticle and high sugar content hinder moisture diffusion under conventional drying (Yildiz and İzli 2019).

### 3.2 Physical, Textural, and Structural Properties and SMER Values of Pre‐Dried Grapes

**FIGURE 3:**
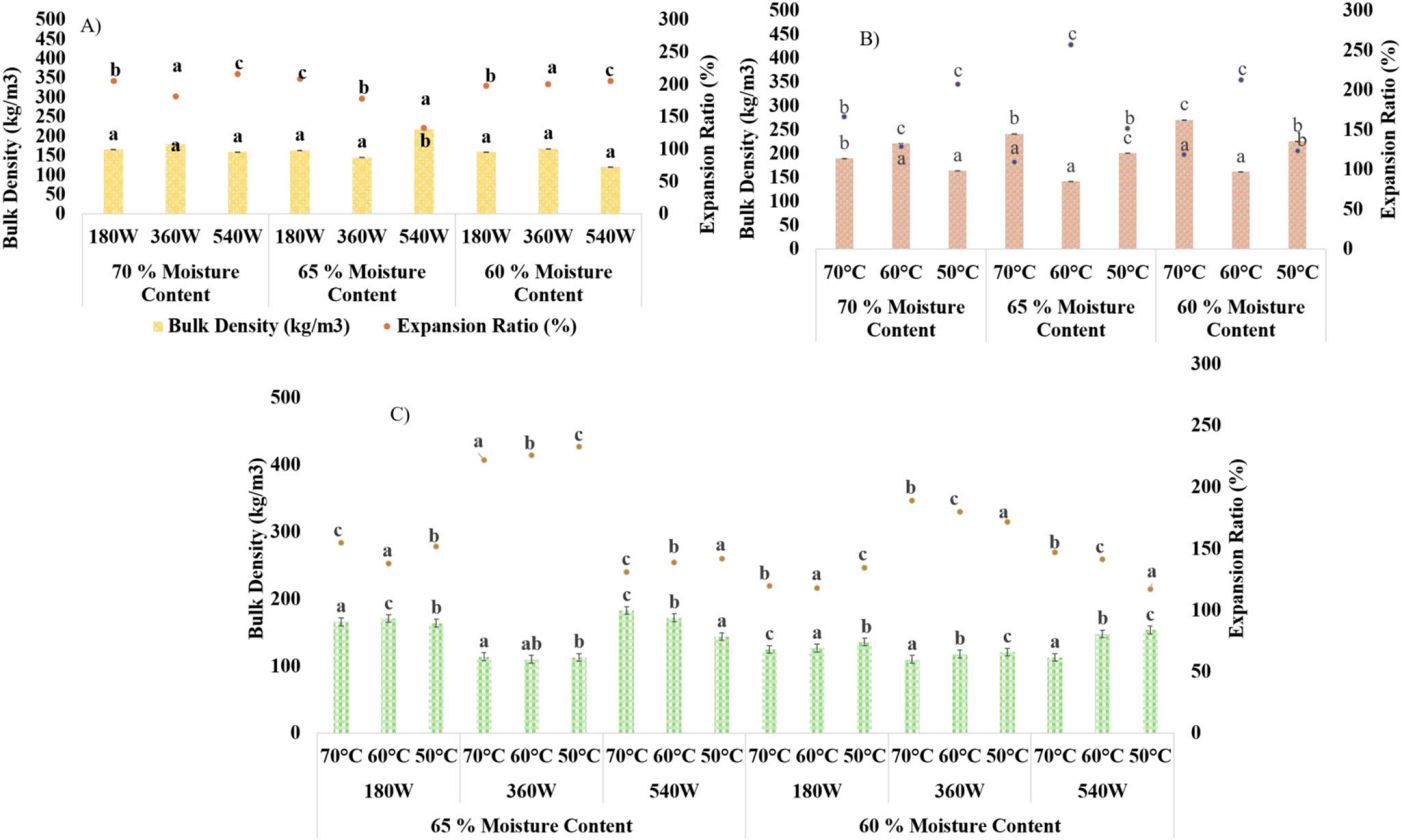
Bulk density and expansion ratio (ER) of pre‐dried grapes with different drying methods followed by explosion puff drying. (A) MD, (B) HAD, and (C) hybrid pre‐drying methods. Different superscript letters indicate significant differences (*p* < 0.05) among drying treatments within the same moisture content.

Tables 3 and 4 present a detailed evaluation of the physical and textural properties of pre‐dried grapes, whereas Figure 3 illustrates the variations in bulk density and ER across different drying methods (MD, HAD, and MD + HAD), followed by EPD. These parameters are essential for assessing the structural and sensory quality of dried products, as they directly influence texture, appearance, and consumer acceptability.

**TABLE 3:** Physical and textural properties (water activity, hardness, crispness, and adhesiveness) of grapes dried with microwave, hot air, and hybrid methods followed by EPD.

	*Microwave drying*					
Moisture content (%)	Microwave power (W)	Water activity	Moisture content (%) (wb)	Hardness (N)	Adhesiveness (N s)	Crispness
70	180	0.455^a^ ± 0.352	5.01^c^ ± 0.151	43.30^a^ ± 1.05	−3.31^a^ ± 0.96	5.5^b^ ± 0.1
	360	0.326^a^ ± 0.125	3.77^b^ ± 0.135	43.31^a^ ± 0.95	−3.09^ab^ ± 1.01	6.6^c^ ± 0.1
	540	0.367^a^ ± 0.240	3.42^a^ ± 0.095	44.29^a^ ± 1.06	−2.29^b^ ± 0.96	5.0^a^ ± 0.1
65	180	0.392^a^ ± 0.175	5.61^c^ ± 0.230	17.28^b^ ± 0.89	−1.75^a^ ± 2.10	12.4^c^ ± 0.0
	360	0.369^a^ ± 0.235	4.88^b^ ± 0.310	15.99^a^ ± 1.02	−0.91^a^ ± 1.95	24.0^b^ ± 0.2
	540	0.357^a^ ± 0.114	4.73^a^ ± 0.130	28.08^c^ ± 1.52	−1.83^a^ ± 2.17	11.6^a^ ± 0.1
60	180	0.389^b^ ± 0.081	5.32^b^ ± 0.322	39.91^b^ ± 1.18	−3.88^a^ ± 1.17	11.7^b^ ± 0.2
	360	0.376^b^ ± 0.102	4.32^a^ ± 0.109	17.63^a^ ± 0.63	−1.30^b^ ± 0.98	29.6^c^ ± 0.1
	540	0.326^a^ ± 0.021	5.81^c^ ± 0.114	17.91^a^ ± 0.99	−0.90^b^ ± 0.56	5.1^a^ ± 0.1

*Hot air drying*						
	Air temperature (°C)	Water activity	Moisture content (%) (wb)	Hardness (N)	Adhesiveness (N s)	Crispness
70	70	0.383^b^ ± 0.001	4.29^b^ ± 0.01	43.30^a^ ± 1.05	−3.31^a^ ± 0.96	5.5^b^ ± 0.1
	60	0.368^a^ ± 0.002	4.02^a^ ± 0.01	43.31^a^ ± 0.95	−3.09^ab^ ± 1.01	6.6^c^ ± 0.1
	50	0.386^b^ ± 0.003	6.08^c^ ± 0.02	44.29^a^ ± 1.06	−2.29^b^ ± 0.96	5.0^a^ ± 0.1
65	70	0.528^c^ ± 0.006	4.08^b^ ± 0.05	17.28^b^ ± 0.89	−1.75^a^ ± 2.10	12.4^c^ ± 0.0
	60	0.306^a^ ± 0.003	3.77^a^ ± 0.01	15.99^a^ ± 1.02	−0.91^a^ ± 1.95	24.0^b^ ± 0.2
	50	0.468^b^ ± 0.006	5.65^c^ ± 0.07	28.08^c^ ± 1.52	−1.83^a^ ± 2.17	11.6^a^ ± 0.1
60	70	0.393^b^ ± 0.014	4.86^c^ ± 0.04	17.91^a^ ± 0.99	−0.90^b^ ± 0.56	5.1^a^ ± 0.1
	60	0.379^b^ ± 0.003	4.46^b^ ± 0.03	17.63^a^ ± 0.63	−1.30^b^ ± 0.98	29.6^c^ ± 0.1
	50	0.363^a^ ± 0.002	2.94^a^ ± 0.03	39.91^b^ ± 1.18	−3.88^a^ ± 1.17	11.7^b^ ± 0.2

	*Hybrid drying*						
	Microwave power (W)	Air temperature (°C)	Water activity	Moisture content (%) (wb)	Hardness (N)	Adhesiveness (N s)	Crispness
65	180	70	0.446^c^ ± 0.004	87.48^b^ ± 0.02	6.88^a^ ± 0.00	−2.67^a^ ± 0.00	4.33^a^ ± 0.02
		60	0.314^a^ ± 0.003	87.84^c^ ± 0.01	24.84^c^ ± 0.00	−2.06^b^ ± 0.04	28.10^c^ ± 0.07
		50	0.415^b^ ± 0.007	82.72^a^ ± 0.01	20.93^b^ ± 0.01	−0.98^c^ ± 0.63	8.15^b^ ± 0.07
	360	70	0.449^c^ ± 0.003	86.78^a^ ± 0.06	15.01^a^ ± 0.02	−1.33^a^ ± 0.01	2.33^a^ ± 0.02
		60	0.316^a^ ± 0.003	88.52^c^ ± 0.02	28.07^b^ ± 0.00	−1.27^a^ ± 0.00	30.10^c^ ± 0.07
		50	0.417^b^ ± 0.001	87.18^b^ ± 0.06	40.50^c^ ± 0.00	−0.42^b^ ± 0.27	4.20^b^ ± 0.11
	540	70	0.392^b^ ± 0.007	88.56^b^ ± 0.04	10.17^a^ ± 0.02	−1.50^b^ ± 0.00	12.15^b^ ± 0.07
		60	0.358^a^ ± 0.002	87.90^a^ ± 0.04	28.05^c^ ± 0.04	−0.40^c^ ± 0.26	14.10^c^ ± 0.08
		50	0.415^c^ ± 0.004	88.93^c^ ± 0.02	12.57^b^ ± 0.00	−3.27^a^ ± 0.00	3.33^a^ ± 0.02
60	180	70	0.434^c^ ± 0.011	85.42^a^ ± 0.01	23.26^b^ ± 0.00	−0.40^c^ ± 0.00	5.33^a^ ± 0.02
		60	0.352^a^ ± 0.002	87.52^b^ ± 0.02	21.80^a^ ± 0.00	−0.56^b^ ± 0.36	15.5^c^ ± 0.14
		50	0.373^b^ ± 0.001	91.45^c^ ± 0.04	39.24^c^ ± 0.00	−1.09^a^ ± 0.00	6.67^b^ ± 0.04
	360	70	0.356^b^ ± 0.001	88.42^b^ ± 0.02	51.24^c^ ± 0.02	−0.79^c^ ± 0.01	7.10^a^ ± 0.14
		60	0.309^a^ ± 0.003	88.45^b^ ± 0.04	18.47^a^ ± 0.00	−2.16^a^ ± 0.00	28.15^c^ ± 0.07
		50	0.386^c^ ± 0.001	87.21^a^ ± 0.04	31.85^b^ ± 0.00	−1.27^b^ ± 0.00	11.33^b^ ± 0.02
	540	70	0.415^c^ ± 0.004	87.06^a^ ± 0.04	13.59^a^ ± 0.01	−0.70^c^ ± 0.01	8.52^a^ ± 0.21
		60	0.393^b^ ± 0.006	89.34^b^ ± 0.03	25.50^b^ ± 0.00	−2.71^b^ ± 0.01	27.33^c^ ± 0.02
		50	0.371^a^ ± 0.004	90.46^c^ ± 0.04	28.66^c^ ± 0.01	−3.20^a^ ± 0.00	15.33^b^ ± 0.02
Raw material		0.162 ± 0.010		82.43 ± 0.07	65.01 ± 0.004	20.12 ± 0.02	—

*Note*: Different letters in the same column are significant at the *p* < 0.05 level.

**TABLE 4:** Statistical analysis of drying method effects on product texture, bulk density, expansion ratio (ER), and specific moisture extraction rate (SMER).

Source	SD	Water activity	Moisture content (%) (wb)	Bulk density (kg/m^3^)	ER (%)	Hardness (N)	Adhesiveness (N s)	Crispness	SMER (kg/kWh)
**Microwave drying**									
Modal	8	0.624	0.001*	0.845	0.003*	0.001*	0.001*	0.005*	0.001*
Intercept	1	0.000	0.000*	0.000*	0.000*	0.000*	0.000*	0.000*	0.000*
*A*	2	0.415	0.000*	0.980	0.000*	0.020*	0.142	0.000*	0.000*
*C*	2	0.421	0.000*	0.543	0.000*	0.001*	0.000*	0.000*	0.001*
*A* × *C*	4	0.659	0.000*	0.662	0.000*	0.002*	0.000*	0.000*	0.005*
*R* ^2^		0.413	0.985	0.298	1.000	0.911	0.994	0.000*	0.988
**Hot air drying**									
Modal	8	0.000*	0.000*	0.000*	0.000*	0.000*	0.000*	0.000*	0.000*
Intercept	1	0.000*	0.000*	0.000*	0.000*	0.000*	0.000*	0.000*	0.000**
*B*	2	0.000*	0.000*	0.000*	0.000*	0.000*	0.000*	0.000*	0.000*
*C*	2	0.000*	0.000*	0.000*	0.000*	0.000*	0.000*	0.000*	0.000*
*B* × *C*	4	0.000*	0.000*	0.000*	0.000*	0.196	0.000*	0.000*	0.000*
*R* ^2^		0.991	0.998	0.984	1.000	0.886	0.999	1.000	0.995
**Hybrid drying**									
Modal	17	0.000*	0.000*	0.000*	0.000*	0.000*	0.000*	0.000*	0.000*
Intercept	1	0.000*	0.000*	0.000*	0.000*	0.000*	0.000*	0.000*	0.000*
*A*	2	0.000*	0.000*	0.000*	0.000*	0.000*	0.000*	0.000*	0.000*
*B*	2	0.000*	0.000*	0.000*	0.000*	0.000*	0.000*	0.000*	0.000*
*C*	1	0.000*	0.000*	0.000*	0.000*	0.000*	0.179	0.000*	0.000*
*A* × *B*	4	0.000*	0.000*	0.000*	0.000*	0.000*	0.000*	0.000*	0.000*
*B* × *C*	2	0.000*	0.000*	0.000*	0.000*	0.000*	0.000*	0.000*	0.451
*A* × *C*	2	0.000*	0.000*	0.000*	0.000*	0.000*	0.000*	0.000*	0.000*
*A* × *B* × *C*	4	0.000*	0.000*	0.000*	0.000*	0.000*	0.000*	0.000*	0.000*
*R* ^2^		0.996	0.998	0.970	1.000	1.000	0.988	1.000	0.971

*Note*: A: Microwave power (W), B: air temperature (°C), C: predicted moisture (%).

*Significant at *p* < 0.05.

The raw grapes, with an initial moisture content of 82.43%, required carefully controlled drying conditions to minimize shrinkage and prevent textural degradation that could compromise product quality. The results indicate that drying conditions, specifically microwave power, air temperature, and initial moisture content, significantly affected water activity, residual moisture, hardness, adhesiveness, crispness, bulk density, ER, and SMER values. Statistical analysis confirmed strong correlations (*R* ^2^ ≥ 0.98 for most properties), underscoring the critical role of these parameters in process optimization.

MD demonstrated high efficiency in moisture removal due to its rapid internal heating mechanism. Increasing microwave power led to lower water activity and moisture levels. However, excessive microwave power increased the risk of local overheating, which may have caused rapid moisture evaporation, cell wall collapse, and structural hardening. These effects likely contributed to reduced brittleness and increased hardness, consistent with previous findings of Darvishi et al. (2013) and Zielinska et al. (2018). In contrast, prolonged HAD at low temperatures (50°C) resulted in excessive shrinkage, higher hardness, and diminished crispness, reflecting the negative effects of extended heat exposure on cell wall integrity and turgor pressure (Wang et al. 2014). These observations highlight the need to balance temperature and drying duration to prevent textural damage while ensuring sufficient dehydration.

At 65% initial moisture content, 360 W microwave power, and 60°C air temperature, MD + HAD yielded superior results in terms of crispness and adhesiveness compared to either method alone. The ER and bulk density values further confirmed that this combined approach minimized shrinkage and structural collapse, thereby enhancing puffing characteristics. Similar to the present findings, Chen et al. (2017) also reported that MD + HAD reduced drying time and energy consumption while maintaining the structural and textural integrity of fruit products.

Lower bulk density values observed in the MD + HAD method, particularly at moderate microwave power (360 W) combined with 60°C hot air, indicate better expansion of grapes during the puff drying process. This suggests that MD + HAD promotes a more favorable internal structure by balancing rapid moisture removal with controlled heat application, thereby preventing excessive cell wall collapse. Similarly, Wang et al. (2023) demonstrated that moderate microwave energy accelerates moisture removal without causing localized overheating, resulting in improved puffing and reduced shrinkage. In contrast, applying MD alone at 65% initial moisture content with high power (540 W) resulted in higher bulk density, reflecting reduced expansion due to localized overheating and excessive moisture loss. In contrast, excessive microwave power (e.g., 540 W) increases the risk of cell wall damage and nonuniform expansion, consistent with Maskan (2000), who found that high‐power MD leads to denser structures due to rapid surface hardening and uneven moisture gradients. As shown in Figure 3, the trend for ER, a key indicator of puffed product quality, was the inverse of bulk density. An increase in ER, associated with greater product volume, corresponded to a decrease in bulk density. The lowest bulk density values and highest ER values were consistently obtained under moderate microwave power and air temperature conditions. Notably, the MD + HAD method produced the highest ER values, confirming its effectiveness in enhancing expansion while minimizing shrinkage.

**FIGURE 4:**
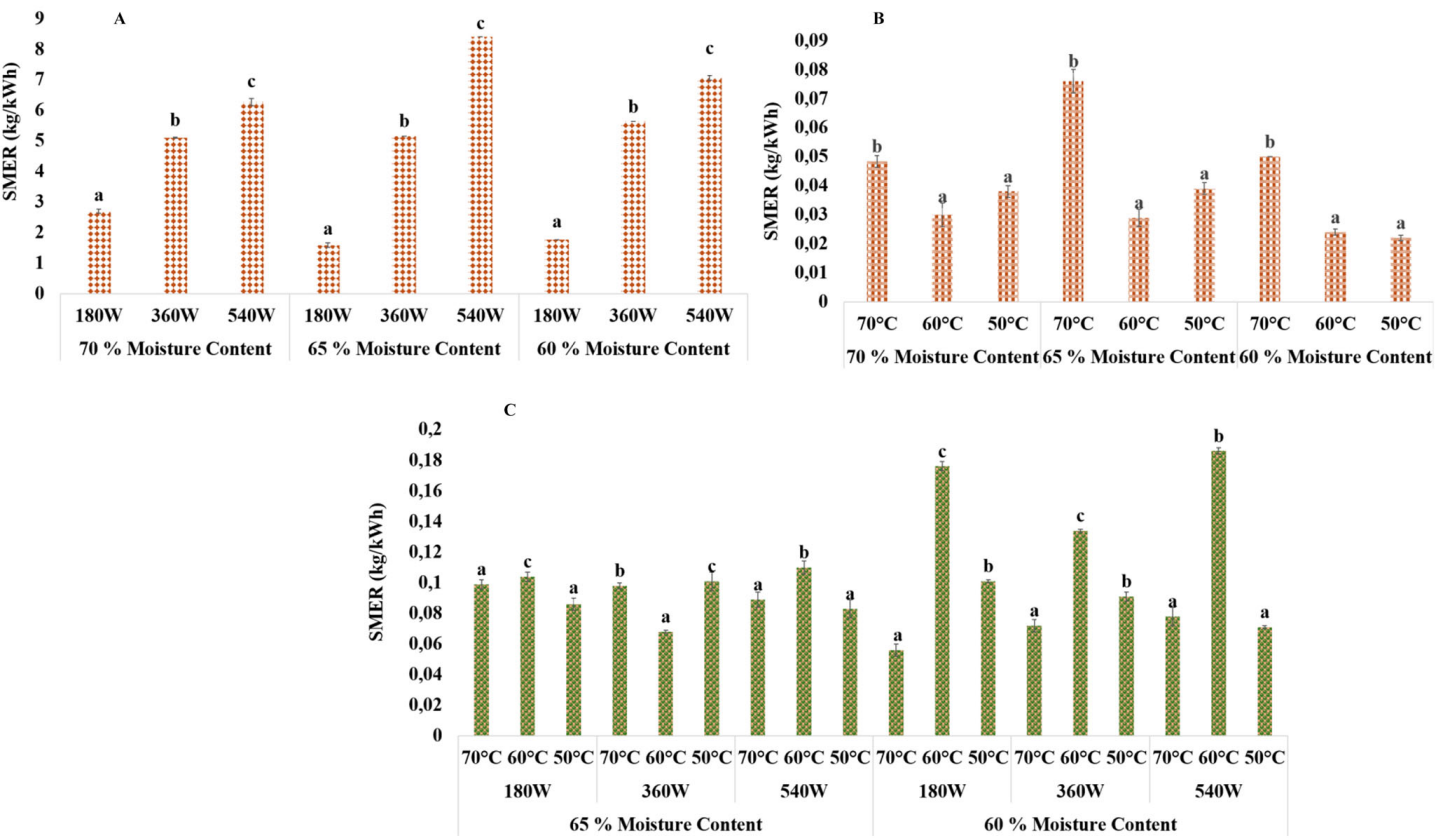
Specific moisture extraction rate (SMER) of dried grape samples with different pre‐drying techniques prior to EPD. (A) Microwave drying, (B) Hot air drying, and (C) Hybrid drying. Different superscript letters indicate significant differences (*p* < 0.05) among drying treatments within the same moisture content.

The statistical analysis (Table 4) further emphasizes the significant influence of drying parameters on bulk density and ER. Interaction effects between factors such as microwave power, air temperature, and initial moisture content were highly significant (*p* < 0.05). The high *R* ^2^ values (e.g., 0.998 for moisture content and 0.970 for bulk density under MD + HAD) demonstrate the strong correlation between drying conditions and product properties. Moreover, the significant interaction terms (AC and BC) highlight that combining moderate microwave power with appropriate air temperature yields the most desirable results. Figure 4 presents the SMER values of grapes dried by MD, HAD, and hybrid pre‐drying methods followed by EPD, further supporting these findings.

SMER, a key indicator of drying efficiency, represents the amount of moisture removed per unit of energy consumed. Among the tested methods, MD generally produced higher SMER values, particularly at elevated microwave powers (e.g., 540 W). An et al. (2024) and Wang et al. (2023) reported that MD significantly increases energy efficiency by directly exciting water molecules through dipole rotation and converting electromagnetic energy into heat within the material, reducing both drying time and energy losses, thus resulting in significantly higher SMER values. In contrast, HAD exhibited the lowest SMER values, reflecting its longer drying times and reliance on indirect heat transfer. Similarly, Deng et al. (2018) and Chou and Chua (2001) reported in their studies that traditional HAD showed low SMER values due to long processing times and dependence on convective heat transfer. As expected, the combined MD + HAD method achieved intermediate SMER values, effectively balancing the rapid moisture removal of MD with the uniform heat distribution of HAD.

Table 4 supports these findings with statistical analyses of SMER alongside water activity, moisture content, RR, and textural attributes (hardness, adhesiveness, and crispness). The results confirm that the drying method (*A*), air temperature (*B*), and initial moisture content (*C*), as well as their interactions, significantly affected SMER values (*p* < 0.001 for all methods). The highest SMER values were obtained under MD conditions with higher power levels and intermediate initial moisture contents, highlighting the importance of optimizing both parameters to maximize energy efficiency. Notably, the statistical analysis also revealed strong correlations between SMER and product quality indicators, particularly RR and crispness, in both MD and hybrid methods. This suggests that higher SMER values not only reflect improved energy utilization but also contribute to superior product quality by preserving structural integrity and desirable texture. Table 5 presents the color and sensory properties of grapes dried by MD, HAD, and hybrid pre‐drying methods followed by EPD, whereas Table 6 summarizes the statistical analyses of these attributes.

**TABLE 5:** Color and sensory evaluation results of grapes dried using microwave and hot air pre‐treatments prior to explosion puff drying.

	*Microwave drying*									
Moisture content (%)	Microwave power (W)	L*	a*	b*	BI	Δ*E*	Color	Adhesiveness	Crispness	General appreciation
70	180	46.72^a^ ± 0.00	2.28^c^ ± 0.00	16.82^b^ ± 0.00	49.08^b^ ± 0.01	44.16^b^ ± 0.00	4.33^a^ ± 0.49	2.91^a^ ± 1.22	2.58^a^ ± 0.79	3.21^a^ ± 1.03
	360	46.39^c^ ± 0.21	0.74^a^ ± 0.87	16.33^a^ ± 0.66	43.45^a^ ± 3.92	46.39^c^ ± 1.63	4.08^a^ ± 0.90	2.83^a^ ± 1.11	2.75^a^ ± 0.97	3.04^a^ ± 0.84
	540	45.95^b^ ± 0.00	1.51^b^ ± 0.01	17.69^c^ ± 0.01	52.07^c^ ± 0.00	44.05^a^ ± 0.00	4.00^a^ ± 0.67	2.90^a^ ± 0.99	3.00^a^ ± 0.82	3.30^a^ ± 0.48
65	180	46.08^c^ ± 1.88	1.97^a^ ± 0.73	17.23^c^ ± 1.90	48.98^b^ ± 2.81	44.09^a^ ± 2.01	4.29^a^ ± 0.54	3.92^a^ ± 1.24	4.25^ab^ ± 0.62	4.00^a^ ± 0.77
	360	43.41^b^ ± 0.46	2.99^b^ ± 1.57	14.54^a^ ± 0.44	45.01^a^ ± 4.87	45.93^c^ ± 0.74	4.10^a^ ± 0.74	3.30^a^ ± 0.95	4.40^b^ ± 0.97	4.05^a^ ± 0.76
	540	42.76^a^ ± 0.06	5.21^c^ ± 1.61	15.16^b^ ± 0.33	51.89^c^ ± 3.98	46.87^b^ ± 0.89	3.94^a^ ± 0.73	3.33^a^ ± 1.32	3.56^a^ ± 0.88	3.67^a^ ± 0.75
60	180	43.85^b^ ± 0.66	2.94^b^ ± 0.13	14.69^b^ ± 0.16	46.26^b^ ± 1.15	47.13^a^ ± 0.28	4.10^c^ ± 0.70	3.50^b^ ± 1.20	2.80^c^ ± 1.47	3.65^c^ ± 0.63
	360	43.35^c^ ± 0.46	1.66^a^ ± 1.57	14.00^a^ ± 0.44	40.93^a^ ± 4.87	47.94^a^ ± 0.74	3.89^b^ ± 1.36	3.22^a^ ± 1.30	2.11^a^ ± 0.93	2.94^b^ ± 1.18
	540	41.89^a^ ± 1.04	3.12^c^ ± 1.03	14.73^b^ ± 0.52	47.88^c^ ± 4.91	47.52^a^ ± 0.30	2.00^a^ ± 1.05	3.60^c^ ± 0.84	2.50^b^ ± 1.27	2.65^a^ ± 0.82

	*Hot air drying*									
	Air temperature (°C)	L*	a*	b*	BI	Δ*E*	Color	Adhesiveness	Crispness	General appreciation
70	70	40.19^c^ ± 0.02	4.37^b^ ± 0.37	12.10^a^ ± 0.41	46.60^b^ ± 0.03	49.26^a^ ± 0.04	3.44^a^ ± 1.13	2.44^a^ ± 1.42	2.00^b^ ± 0.87	2.72^a^ ± 1.18
	60	38.79^a^ ± 0.03	4.55^c^ ± 0.02	12.61^b^ ± 0.01	46.96^c^ ± 2.11	50.26^b^ ± 0.03	3.33^a^ ± 0.78	3.42^a^ ± 0.67	4.25^c^ ± 0.75	3.75^a^ ± 0.50
	50	40.10^b^ ± 0.04	3.41^a^ ± 0.01	11.93^a^ ± 0.01	40.91^a^ ± 0.03	50.43^c^ ± 0.02	3.00^a^ ± 1.50	2.22^a^ ± 1.09	1.33^a^ ± 0.50	2.44^a^ ± 1.13
65	70	42.98^c^ ± 0.02	3.20^a^ ± 0.01	15.07^c^ ± 0.05	47.85^c^ ± 0.02	46.67^a^ ± 0.05	3.20^a^ ± 1.48	3.20^a^ ± 1.23	2.00^b^ ± 0.94	3.20^a^ ± 0.89
	60	42.74^b^ ± 0.01	4.34^c^ ± 0.03	14.00^b^ ± 0.01	46.48^b^ ± 0.02	47.10^b^ ± 0.05	3.56^a^ ± 0.83	2.50^a^ ± 1.42	2.00^b^ ± 1.00	2.61^a^ ± 0.79
	50	40.29^a^ ± 0.03	3.63^b^ ± 0.02	12.17^a^ ± 0.01	42.02^a^ ± 0.01	50.04^c^ ± 0.03	3.11^a^ ± 1.45	2.56^a^ ± 1.13	1.22^a^ ± 0.44	2.72^a^ ± 1.03
60	70	38.98^a^ ± 0.02	3.57^a^ ± 0.04	9.20^a^ ± 0.01	43.10^c^ ± 0.07	53.00^c^ ± 0.71	3.60^a^ ± 1.35	2.80^a^ ± 1.32	3.20^a^ ± 1.23	3.40^a^ ± 0.84
	60	41.26^b^ ± 0.03	3.87^b^ ± 0.01	14.05^b^ ± 0.04	40.71^a^ ± 0.01	48.07^b^ ± 0.01	3.10^a^ ± 1.52	2.60^a^ ± 1.07	2.50^a^ ± 0.97	3.05^a^ ± 1.01
	50	41.26^b^ ± 0.04	4.26^c^ ± 0.08	14.26^c^ ± 0.03	41.26^b^ ± 0.01	41.26^a^ ± 0.04	3.10^a^ ± 1.52	2.20^a^ ± 1.32	2.00^a^ ± 1.33	2.60^a^ ± 1.17

	*Hybrid drying*										
	Microwave power (W)	Air temperature (°C)	L*	a*	b*	BI	Δ*E*	Color	Adhesiveness	Crispness	General appreciation
65	180	70	43.33^a^ ± 0.02	2.48^a^ ± 0.02	14.95^a^ ± 0.04	45.53^a^ ± 0.02	46.91^c^ ± 0.03	4.33^a^ ± 0.65	3.25^a^ ± 1.14	2.00^a^ ± 1.21	2.75^a^ ± 0.84
		60	43.45^b^ ± 0.01	2.93^b^ ± 0.02	15.05^b^ ± 0.04	44.58^b^ ± 0.02	45.56^b^ ± 0.04	4.56^a^ ± 0.53	4.11^a^ ± 1.05	4.44^b^ ± 0.73	4.33^b^ ± 0.87
		50	42.52^c^ ± 0.01	2.48^a^ ± 0.06	16.66^c^ ± 0.08	49.84^c^ ± 0.03	45.08^a^ ± 0.06	4.11^a^ ± 0.60	4.22^a^ ± 0.44	4.44^b^ ± 0.53	4.22^b^ ± 0.44
	360	70	44.70^b^ ± 0.28	2.46^b^ ± 0.04	15.35^b^ ± 0.04	45.28^a^ ± 0.06	45.92^b^ ± 0.02	4.10^a^ ± 0.57	3.40^a^ ± 0.97	2.40^a^ ± 0.97	3.50^a^ ± 0.47
		60	43.07^a^ ± 0.05	1.71^a^ ± 0.01	15.61^c^ ± 0.01	45.88^b^ ± 0.06	47.27^c^ ± 0.05	4.11^a^ ± 0.78	4.11^a^ ± 0.93	4.56^b^ ± 0.53	4.17^b^ ± 0.61
		50	43.06^a^ ± 0.04	3.30^c^ ± 0.21	14.50^a^ ± 0.04	48.94^c^ ± 0.01	44.81^a^ ± 0.01	4.40^a^ ± 0.70	4.00^a^ ± 1.05	4.80^b^ ± 0.42	4.80^c^ ± 0.42
	540	70	43.69^b^ ± 0.06	1.62^a^ ± 0.02	13.68^a^ ± 0.02	39.46^a^ ± 0.04	48.01^c^ ± 0.17	4.40^b^ ± 0.52	3.40^a^ ± 0.70	2.40^a^ ± 0.97	3.60 ± 0.52
		60	44.32^c^ ± 0.04	1.78^b^ ± 0.06	14.41^b^ ± 0.01	41.49^b^ ± 0.06	47.14^b^ ± 0.03	3.83^a^ ± 0.39	4.25^b^ ± 0.97	4.33^b^ ± 0.78	4.38 ± 0.71
		50	43.54^a^ ± 0.03	2.41^c^ ± 0.01	16.50^c^ ± 0.28	50.61^c^ ± 0.01	45.73^a^ ± 0.09	4.04^ab^ ± 0.62	4.58^b^ ± 0.51	4.50^b^ ± 0.67	4.42 ± 0.47
60	180	70	43.54^b^ ± 0.03	2.91^b^ ± 0.04	14.53^a^ ± 0.02	44.67^a^ ± 0.05	46.90^c^ ± 0.04	4.30^a^ ± 0.48	3.30^a^ ± 1.16	3.00^a^ ± 0.94	3.85^a^ ± 0.58
		60	43.06^a^ ± 0.04	3.95^c^ ± 0.07	16.82^b^ ± 0.01	55.18^c^ ± 0.02	45.06^b^ ± 0.04	4.44^a^ ± 0.53	4.33^a^ ± 0.87	4.78^b^ ± 0.44	4.44^a^ ± 0.73
		50	44.92^c^ ± 0.01	2.43^a^ ± 0.02	16.82^b^ ± 0.01	49.79^b^ ± 0.03	44.75^a^ ± 0.04	3.75^a^ ± 0.79	4.50^a^ ± 0.52	4.83^b^ ± 0.40	4.13^a^ ± 0.64
	360	70	43.84^a^ ± 0.03	2.97^a^ ± 0.05	16.49^b^ ± 0.03	50.93^b^ ± 0.02	45.32^b^ ± 0.12	4.30^a^ ± 0.48	3.70^a^ ± 0.95	2.80^a^ ± 1.03	3.55^a^ ± 0.50
		60	44.66^b^ ± 0.07	3.34^b^ ± 0.03	15.23^a^ ± 0.02	46.38^a^ ± 0.06	45.61^c^ ± 0.01	3.60^a^ ± 0.52	4.00^a^ ± 1.05	4.80^b^ ± 0.42	4.00^a^ ± 0.62
		50	45.41^c^ ± 0.01	4.32^c^ ± 0.01	18.61^c^ ± 0.01	58.37^c^ ± 0.05	42.35^a^ ± 0.04	3.50^ab^ ± 0.89	4.17^a^ ± 1.22	4.75^b^ ± 0.89	4.04^a^ ± 0.89
	540	70	46.36^c^ ± 0.04	1.81^a^ ± 0.01	16.63^b^ ± 0.01	46.42^a^ ± 0.01	44.52^a^ ± 0.01	4.50^b^ ± 0.73	3.60^a^ ± 0.83	3.10^a^ ± 1.39	3.80^ab^ ± 0.67
		60	44.26^a^ ± 0.07	3.58^c^ ± 0.02	16.58^a^ ± 0.04	51.90^c^ ± 0.11	44.76^b^ ± 0.05	4.56^b^ ± 0.53	4.11^a^ ± 1.05	4.44^a^ ± 0.73	4.33^b^ ± 0.87
		50	44.91^b^ ± 0.04	1.91^b^ ± 0.03	17.73^c^ ± 0.06	49.79^b^ ± 0.03	44.75^b^ ± 0.07	3.33^a^ ± 0.71	3.89^a^ ± 0.78	3.22^a^ ± 0.97	3.61^a^ ± 0.70
Raw material			53.13 ± 0.22	−6.30 ± 0.22	22.75 ± 0.17	—	—	—	—	—	—

*Note*: Different letters in the same column are significant at the *p* < 0.05 level.

Abbreviation: BI, browning index.

**TABLE 6:** Statistical analysis results for color parameters and sensory scores under different drying conditions.

Source	SD	L*	a*	b*	BI	Δ*E*	Color	Adhesiveness (N s)	Crispness	General appreciation
**Microwave drying**										
Modal	8	0.000*	0.000*	0.000*	0.000*	0.000*	0.101	0.874	0.120	0.398
Intercept	1	0.000*	0.000*	0.000*	0.000*	0.000*	0.000*	0.000*	0.000*	0.000*
*A*	2	0.000*	0.000*	0.000*	0.000*	0.000*	0.068	0.721	0.908	0.408
*C*	2	0.000*	0.000*	0.000*	0.000*	0.000*	0.172	0.348	0.013	0.089
*A* × *C*	4	0.000*	0.000*	0.000*	0.000*	0.000*	0.177	0.980	0.495	0.387
*R* ^2^		1.000	1.00	0.999	1.000	1.000	0.687	0.277	0.670	0.514
**Hot air drying**										
Modal	8	0.000*	0.000*	0.000*	0.000*	0.000*	1.000	0.9180	0.018	0.804
Intercept	1	0.000*	0.000*	0.000*	0.000*	0.000*	0.000*	0.000*	0.000*	0.000*
*B*	2	0.000*	0.000*	0.000*	0.000*	0.000*	0.875	0.511	0.011	0.502
*C*	2	0.000*	0.000*	0.000*	0.000*	0.000*	0.949	0.892	0.063	0.850
*B* × *C*	4	0.000*	0.000*	0.000*	0.000*	0.000*	0.997	0.871	0.060	0.661
*R* ^2^		1.000	0.920	1.000	0.979	0.995	0.788	0.431	0.628	0.279
**Hybrid drying**										
Modal	17	0.000*	0.000*	0.000*	0.000*	0.000*	0.000*	0.044*	0.000*	0.000*
Intercept	1	0.000*	0.000*	0.000*	0.000*	0.000*	0.000*	0.000*	0.000*	0.000*
*A*	2	0.000*	0.000*	0.000*	0.000*	0.000*	0.121	0.971	0.207	0.855
*B*	2	0.000*	0.000*	0.000*	0.000*	0.000*	0.002*	0.000*	0.000*	0.000*
*C*	1	0.000*	0.000*	0.000*	0.000*	0.000*	0.066	0.591	0.051	0.995
*A* × *B*	4	0.000*	0.000*	0.000*	0.000*	0.000*	0.026*	0.913	0.032*	0.017*
*B* × *C*	2	0.000*	0.000*	0.000*	0.000*	0.000*	0.044*	0.544	0.195	0.036*
*A* × *C*	2	0.000*	0.000*	0.000*	0.000*	0.000*	0.005*	0.747	0.006*	0.002*
*A* × *B* × *C*	4	0.000*	0.000*	0.000*	0.000*	0.000*	0.024*	0.646	0.079	0.562
*R* ^2^		0.997	0.998	0.999	1.000	0.999	0.485	0.236	0.803	0.625

*Note*: A: Microwave power (W), *B*: air temperature (°C), *C*: predicted moisture (%).

Abbreviation: BI, browning index.

*Significant at *p* < 0.05.

**FIGURE 5:**
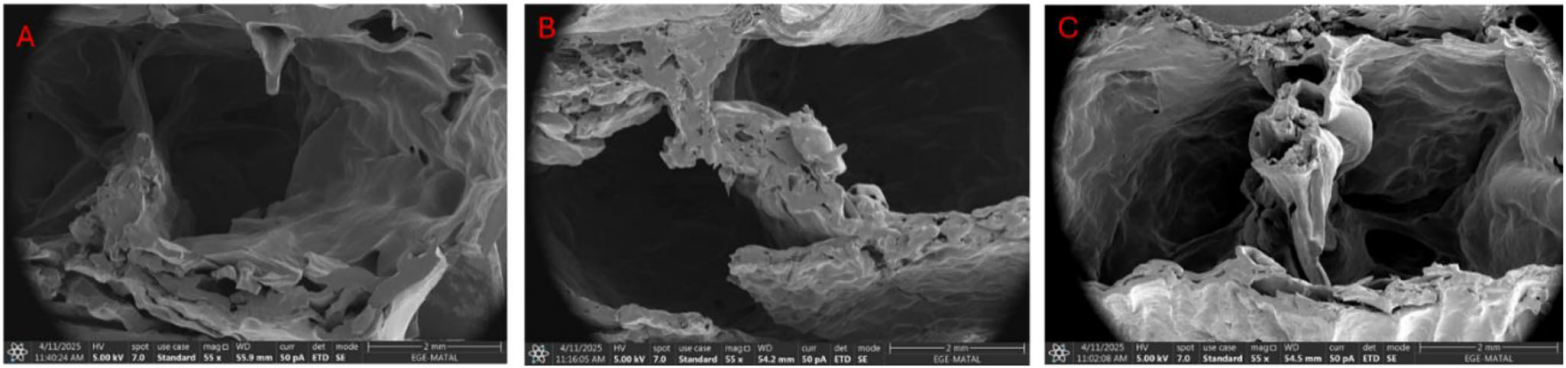
SEM images of grapes pre‐dried with (A) MD 360 W, 70% moisture content; (B) HAD 60°C, 65% moisture content; and (C) combination of these two methods (MD + HAD) and then dried with explosion puff drying method.

MD at higher initial moisture contents (65%–70%) and lower power levels (180 W) generally preserved color more effectively, as indicated by higher L* values. In contrast, high microwave power (540 W) significantly increased the BI and total color difference (Δ*E*), reflecting greater nonenzymatic browning, particularly Maillard reactions. Sensory evaluation of MD samples showed moderate scores for overall appreciation and crispness, with noticeable declines at higher power levels, suggesting that excessive drying intensity negatively affects sensory quality. In the hybrid drying method (MD + HAD), the combination of microwave power and air temperature exhibited synergistic effects. Grapes pre‐dried at moderate air temperatures (60°C) and microwave power (180–360 W) showed lower BI values and higher sensory scores, particularly for crispness and overall impression, consistent with findings in carrot and green tea studies where hybrid drying maintained desirable color and texture (Jin et al. 2018). Conversely, high air temperature (70°C) combined with high microwave power (540 W) caused overdrying, diminished sensory acceptance, and a duller product appearance. Statistical analysis (Table 6) confirmed that microwave power (*A*), air temperature (*B*), and moisture content (*C*), along with their interactions, significantly affected color and sensory attributes (*p < *0.001). Parameters, such as L*, a*, b*, and Δ*E*, were all highly dependent on drying intensity. Notably, the MD + HAD method under moderate conditions (360 W, 60°C) yielded the highest overall appreciation scores (*p* < 0.05), highlighting the role of optimized conditions in preserving both visual and sensory quality. Overall, MD + HAD was identified as the most effective pre‐drying method, combining improved drying rate (ER), crispness, L* value, antioxidant activity (DPPH), sensory performance (overall impression), and energy efficiency (SMER). The optimal pre‐drying conditions were determined as MD at 360 W until 70% moisture content, followed by HAD at 60°C until 65% moisture content. Grapes pre‐dried under these conditions exhibited the highest crispness, lowest adhesiveness, and favorable water activity, ensuring both desirable texture and stability. Supporting evidence from SEM (Figure 5) illustrates the microstructural differences among grapes pre‐dried at 70% moisture with 360 W MD, at 65% with 60°C HAD, and under the optimized hybrid conditions. These microstructural insights, combined with quality and efficiency data, guided the subsequent optimization of puff drying parameters using grapes pre‐dried under the identified optimal conditions.

In Image A, the internal structure is highly porous and irregular, with large cavities and collapsed cell walls. This indicates that MD caused rapid internal heating and vaporization, leading to structural breakdown. The uneven distribution of microwave energy likely promoted localized overheating and nonuniform pore development. In Image B, the structure appears denser and more compact, with limited porosity. Pronounced cellular collapse and shrinkage reflect a slower moisture removal process dominated by surface evaporation, characteristic of HAD. Although this method preserves cell wall integrity better than MD, it fails to generate the open, porous structure desirable for puffing. In Image C, representing the hybrid MD + HAD method, the structure displays a more homogeneous and well‐developed porous network. Expanded regions with reduced collapse suggest that the combined effects of volumetric heating from MD and controlled surface drying from HAD create a balanced environment, resulting in improved structural integrity and puffing potential.

### 3.3 Optimization of Explosion Puff Drying Conditions

**FIGURE 6:**
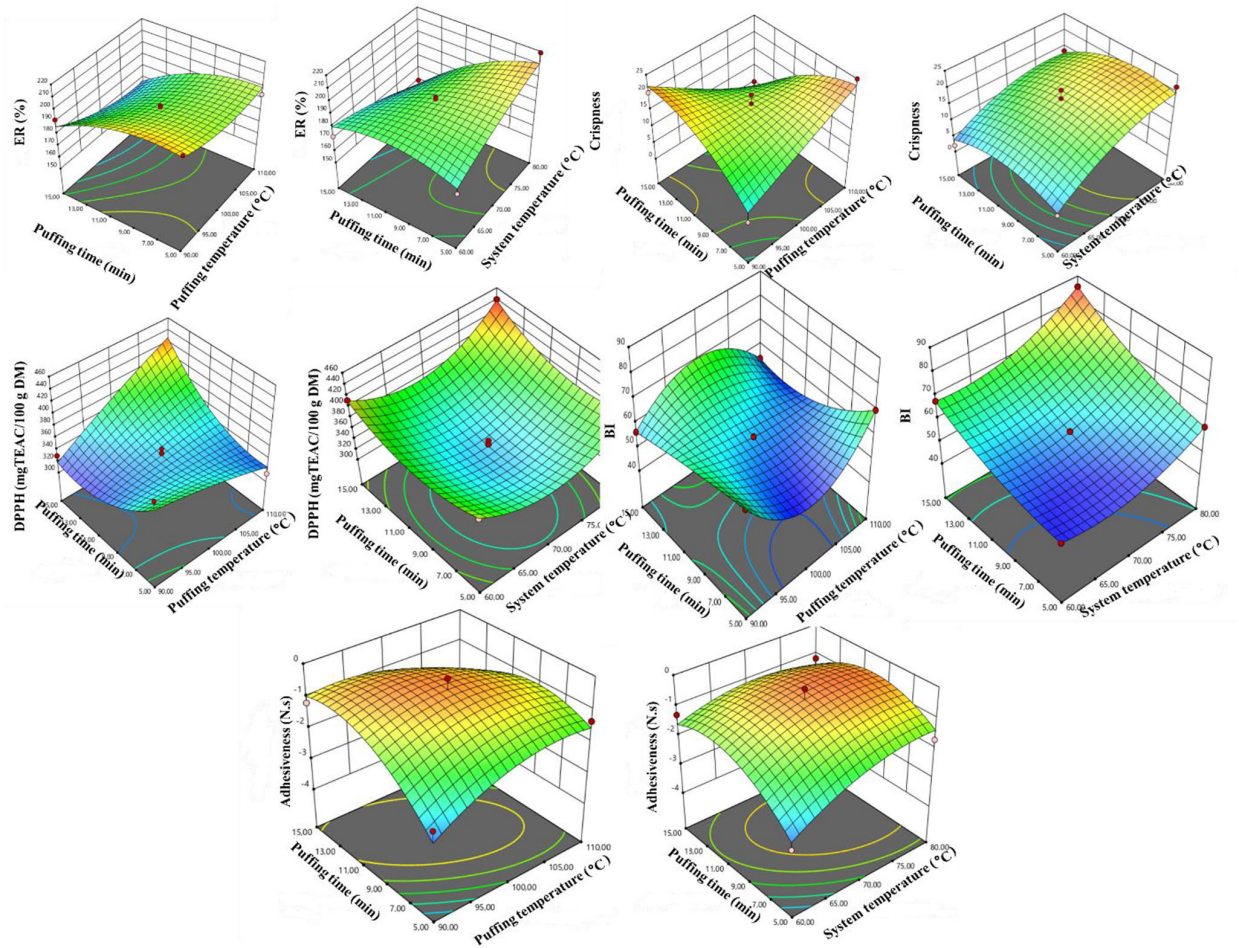
Contour maps showing the effects of independent variables on response variable. ER, expansion ratio; TEAC, Trolox equivalent antioxidant capacity.

The experimental design (Table 7), statistical results (Table 8), and 3D response surface plots (Figure 6) provide a comprehensive assessment of the effects of explosion puff drying (EPD) parameters on the physicochemical, structural, and textural properties of pre‐dried grapes. The results emphasize the critical influence of puffing temperature, puffing time, and system temperature on key quality attributes, including moisture content, ER, bulk density, antioxidant activity, and texture. Response surface methodology (RSM) revealed the complex interactions among process variables, enabling precise optimization of puff drying conditions to enhance both product quality and processing efficiency. The Box–Behnken experimental design applied in this study systematically identified the optimal parameter ranges for achieving desirable physicochemical stability, structural integrity, and textural characteristics in puff‐dried grapes.

**TABLE 7:** The analysis results of the pre‐dried grapes subjected to puff drying according to the Box–Behnken experimental design.

RUN	Puffing temperature (°C) (*X*_1_)	Puffing time, (min) (*X*_2_)	System temperature (°C) (*X*_3_)	FRAP (mg AA/100 g DM)	DPPH (mg TEAC/100 g DM)	ABTS (%)	TPC (mg GAE/100 g DM)	Water activity	Moisture content (%) (wb)	Bulk density (kg/m^3^)	ER (%)
1	100	15	80	467.82 ± 4.76	440.88 ± 4.07	92.26 ± 3.29	603.57 ± 4.37	0.446 ± 4.37	6.17 ± 1.10	213.20 ± 0.02	159.17 ± 0.12
2	100	15	60	432.00 ± 3.31	412.57 ± 0.30	94.93 ± 2.63	629.63 ± 1.88	0.536 ± 4.37	6.47 ± 2.17	206.45 ± 0.01	172.00 ± 1.12
3	90	10	60	419.74 ± 0.50	328.88 ± 5.18	96.91 ± 0.10	374.34 ± 4.95	0.530 ± 4.37	5.98 ± 0.96	204.60 ± 1.19	205.46 ± 0.93
4	110	5	70	382.32 ± 5.79	313.96 ± 5.23	94.36 ± 0.20	317.27 ± 2.05	0.434 ± 4.37	4.53 ± 2.36	199.05 ± 2.22	193.68 ± 0.57
5	90	10	80	486.76 ± 2.39	383.53 ± 0.18	95.46 ± 0.30	609.85 ± 2.55	0.427 ± 4.37	6.25 ± 3.17	201.76 ± 0.90	192.00 ± 1.58
6	110	10	60	455.43 ± 4.22	422.57 ± 2.48	97.06 ± 0.43	605.73 ± 1.71	0.479 ± 4.37	5.62 ± 1.10	188.06 ± 1.11	194.00 ± 2.17
7	90	5	70	448.58 ± 0.10	395.76 ± 6.33	98.77 ± 0.55	659.05 ± 3.01	0.429 ± 4.37	5.64 ± 1.88	188.42 ± 1.57	208.39 ± 3.05
8	100	5	80	420.18 ± 5.62	407.17 ± 1.00	97.84 ± 0.43	708.69 ± 0.29	0.438 ± 4.37	4.07 ± 0.97	168.87 ± 0.96	219.00 ± 2.56
9	100	10	70	406.80 ± 1.62	349.49 ± 4.81	97.63 ± 0.16	543.51 ± 2.56	0.466 ± 4.37	5.58 ± 1.53	196.71 ± 2.10	191.43 ± 4.10
10	110	15	70	431.41 ± 7.12	413.50 ± 1.27	98.68 ± 0.04	650.07 ± 3.16	0.500 ± 4.37	6.72 ± 2.09	209.48 ± 3.35	164.89 ± 2.17
11	110	10	80	469.47 ± 1.93	396.34 ± 1.72	94.89 ± 0.01	732.48 ± 3.98	0.463 ± 4.37	6.21 ± 4.37	209.95 ± 2.15	191.43 ± 3.58
12	100	10	70	430.20 ± 7.34	342.00 ± 6.20	97.87 ± 0.16	587.40 ± 5.34	0.462 ± 4.37	5.38 ± 4.52	255.59 ± 0.97	192.00 ± 2.83
13	100	10	70	437.35 ± 2.59	345.17 ± 1.45	98.57 ± 0.04	585.49 ± 1.51	0.461 ± 4.37	5.97 ± 0.28	252.80 ± 2.11	194.00 ± 2.59
14	100	5	60	407.86 ± 4.41	402.98 ± 2.96	98.54 ± 0.13	568.18 ± 4.70	0.466 ± 4.37	5.97 ± 0.21	185.73 ± 1.53	173.33 ± 1.54
15	90	15	70	402.57 ± 4.76	323.36 ± 4.62	96.41 ± 0.06	539.17 ± 1.11	0.459 ± 4.37	4.02 ± 0.36	183.90 ± 0.90	190.00 ± 2.96
16	100	10	70	400.23 ± 3.59	326.46 ± 6.82	98.13 ± 0.11	540.57 ± 5.40	0.460 ± 4.37	4.33 ± 0.29	187.67 ± 1.56	193.10 ± 0.87
17	100	10	70	401.10 ± 0.53	321.57 ± 2.95	97.73 ± 0.35	541.03 ± 1.88	0.461 ± 4.37	4.89 ± 0.26	185.67 ± 4.37	192.50 ± 0.57

RUN	Puffing temperature (°C) (*X*_1_)	Puffing time, (min) (*X*_2_)	System temperature (°C) (*X*_3_)	Hardness (N)	Adhesiveness (N s)	Crispness	L*	a*	b*	BI	Δ*E*
1	100	15	80	53.93 ± 0.20	−1.17 ± 0.23	14 ± 0.22	38.69 ± 4.86	4.06 ± 0.87	21.55 ± 4.71	85.58 ± 0.56	44.65 ± 5.97
2	100	15	60	14.62 ± 0.17	−1.29 ± 0.19	2 ± 0.02	36.05 ± 3.43	2.30 ± 0.97	17.30 ± 2.26	67.83 ± 1.97	49.63 ± 3.64
3	90	10	60	15.98 ± 0.18	−1.87 ± 0.22	11 ± 0.29	39.80 ± 2.94	3.08 ± 0.97	17.86 ± 2.92	65.11 ± 1.10	46.76 ± 2.10
4	110	5	70	35.57 ± 0.86	−1.35 ± 0.25	25 ± 0.19	41.36 ± 3.97	1.96 ± 0.76	20.46 ± 3.21	70.08 ± 1.72	44.61 ± 3.90
5	90	10	80	30.79 ± 0.18	−1.35 ± 0.06	25 ± 0.59	36.91 ± 1.30	5.85 ± 1.24	17.34 ± 2.73	74.57 ± 4.85	47.72 ± 1.71
6	110	10	60	24.68 ± 0.87	−2.06 ± 0.19	8 ± 0.87	39.17 ± 2.21	1.85 ± 0.61	17.37 ± 3.00	61.81 ± 4.96	47.94 ± 2.55
7	90	5	70	24.82 ± 0.59	−2.43 ± 0.35	5 ± 0.02	39.72 ± 1.18	3.42 ± 1.01	18.96 ± 0.64	69.38 ± 1.58	45.86 ± 0.35
8	100	5	80	29.01 ± 0.52	−1.70 ± 0.39	22 ± 0.09	38.43 ± 3.22	3.13 ± 0.69	16.46 ± 1.42	61.33 ± 2.99	48.43 ± 2.58
9	100	10	70	25.09 ± 0.48	−0.86 ± 0.47	18 ± 0.12	41.08 ± 4.72	1.80 ± 0.55	17.52 ± 2.68	57.06 ± 3.63	46.74 ± 4.82
10	110	15	70	35.90 ± 0.46	−2.63 ± 0.94	5 ± 40.09	39.49 ± 1.80	1.57 ± 0.58	16.07 ± 1.76	54.15 ± 4.01	48.69 ± 2.14
11	110	10	80	30.50 ± 0.62	−0.85 ± 0.46	14 ± 0.22	38.62 ± 1.26	3.75 ± 0.54	16.02 ± 0.53	59.51 ± 0.59	48.38 ± 0.56
12	100	10	70	27.47 ± 4.37	−0.25 ± 0.93	15 ± 0.59	40.19 ± 0.79	2.44 ± 0.23	16.73 ± 1.03	56.92 ± 2.80	47.39 ± 1.01
13	100	10	70	27.47 ± 0.20	−0.25 ± 0.22	15 ± 0.96	40.19 ± 2.84	2.44 ± 0.22	16.73 ± 2.10	56.92 ± 6.35	47.39 ± 2.76
14	100	5	60	20.25 ± 0.17	−3.02 ± 0.46	4 ± 0.03	40.36 ± 0.85	1.40 ± 1.68	15.82 ± 1.75	50.92 ± 7.37	48.44 ± 1.58
15	90	15	70	29.50 ± 0.12	−1.19 ± 0.18	20 ± 1.10	41.16 ± 3.17	2.15 ± 0.20	15.54 ± 2.86	53.27 ± 6.19	46.22 ± 3.74
16	100	10	70	27.39 ± 0.08	−0.80 ± 0.65	20 ± 0.99	40.23 ± 0.89	2.07 ± 0.70	16.84 ± 1.93	55.55 ± 5.66	47.19 ± 1.78
17	100	10	70	28.43 ± 2.10	−0.80 ± 0.48	20 ± 1.10	42.57 ± 1.50	2.13 ± 0.67	17.33 ± 0.75	57.59 ± 3.58	46.85 ± 1.32

Abbreviations: BI, browning index; DM, dry matter; FRAP, ferric reducing antioxidant potential; GAE, gallic acid equivalent; TEAC, Trolox equivalent antioxidant capacity; TPC, total phenolic content.

**TABLE 8:** Analysis of variance (ANOVA) evaluation of linear quadratic and interaction terms for each response variables of Puff dried sultana grapes.

*Source*	*FRAP (mg AA/100 g DM)*		*DPPH (TEAC/100 g DM)*		*ABTS (%)*		*TPC (mg GAE/100 g DM)*		*Water activity*		*Moisture content (%) (wb)*		*Bulk density (kg/m^3^)*		*ER (%)*	
	SS	*p* value	SS	*p* value	SS	*p* value	SS	*p* value	SS	*p* value	SS	*p* value	SS	*p* value	SS	*p* value
*Model*	10,708.5	0.0288	25,348.31	0.001	34.89	0.3885	70,721.0	0.0133	0.0151	0.0002	8.95	0.6064	0.0024	0.9410	3453.47	<0.0001*
*X_1_*	59.50	0.6421	1469.71	0.042	1.88	0.4594	169.15	0.7283	0.0001	0.2504	0.1499	0.7326	0.0001	0.7551	769.91	<0.0001*
*X_2_*	753.08	0.1326	737.98	0.122	4.36	0.2723	50.83	0.8485	0.0039	0.0002	0.6413	0.4860	0.0007	0.3959	1109.97	<0.0001*
*X_3_*	2086.06	0.0253	463.92	0.206	6.10	0.2017	7275.05	0.0495	0.0071	0.0001	0.6845	0.4722	0.0000	0.9145	107.49	0.0012
*X_1_X_2_*	2129.66	0.0243	6850.49	0.001	7.20	0.1697	40,911.1	0.0008	0.0003	0.0809	3.48	0.1305	0.0000	0.8233	38.43	0.0166
*X_1_X_3_*	701.47	0.1446	1635.52	0.034	0.1292	0.8434	742.61	0.4733	0.0019	0.0014	0.0248	0.8891	0.0002	0.6767	33.10	0.0228
*X_2_X_3_*	138.15	0.4899	145.51	0.461	0.9636	0.5930	6936.83	0.0537	0.0010	0.0080	1.70	0.2705	0.0001	0.6905	979.97	<0.0001*
*X_1_^2^*	779.13	0.1271	74.79	0.593	0.2371	0.7892	1650.67	0.2958	8.25	0.7443	0.0149	0.9139	0.0001	0.6894	127.80	0.0007
*X_2_^2^*	634.59	0.1623	3506.08	0.006	0.7547	0.6354	773.74	0.4645	0.0001	0.3089	0.0061	0.9449	0.0007	0.3690	279.97	<0.0001*
*X_3_^2^*	3457.31	0.0082	9880.61	0.000	12.59	0.0827	12,409.6	0.0174	0.0008	0.0116	2.23	0.2123	0.0003	0.5881	22.84	0.0466
*Residual*	1821.47		1678.44		21.52		9052.09		0.0005		8.30		0.0057		27.46	
*Pure error*	713.48		297.97		0.9258		2570.46		0.000		5.68		0.0049		3.83	
*Total*	12,530.0		27,026.7		56.40		79,773.1		0.0156		17.25		0.0080		3480.92	
*R^2^*	0.8546		0.9379		0.6285		0.8865		0.9678		0.6705		0.2935		0.9921	
*R^2^_adj_*	0.6677		0.8581		0.1280		0.7406		0.9265		0.3180		0.6148		0.9820	
C.V.*	3.75		4.15		1.81		6.18		1.82		21.37		14.07		1.04	
A.P*	8.77		10.20		4.58		8.94		18.37		2.78		1.60		36.11	

	Hardness (N)		Adhesiveness (N s)		Crispness		L*		a*		b*		BI		Δ*E*	
*Source*	SS	*p* value	SS	*p* value	SS	*p* value	SS	*p* value	SS	*p* value	SS	*p* value	SS	*p* value	SS	*p* value
*Model*	1109.59	0.1263	8.92	0.0121	774.73	0.0089	31.45	0.0209	17.27	0.0084	9.77	0.9646	614.18	0.0389	16.19	0.5302
*X_1_*	81.73	0.2440	0.0003	0.9686	10.13	0.3941	0.1635	0.6372	4.08	0.0059	0.1472	0.8541	134.35	0.0254	0.7346	0.5497
*X_2_*	73.93	0.2656	0.6206	0.0876	27.50	0.1782	2.62	0.0901	0.0328	0.7373	0.0004	0.9919	34.77	0.1932	0.7766	0.5388
*X_3_*	589.93	0.0112	1.26	0.0256	290.00	0.0018	0.9250	0.2807	8.31	0.0009	1.14	0.6116	38.76	0.1424	1.61	0.3829
*X_1_X_2_*	4.74	0.7682	1.59	0.0156	306.25	0.0016	2.58	0.0920	0.0703	0.6249	1.30	0.5879	33.62	0.1999	2.39	0.2941
*X_1_X_3_*	20.21	0.5471	0.1218	0.4084	16.00	0.2912	1.37	0.1985	0.1949	0.4229	0.1708	0.8430	34.56	0.1944	0.0676	0.8542
*X_2_X_3_*	233.39	0.0687	0.3602	0.1743	8.51	0.4327	5.22	0.0274	0.0001	0.9852	3.27	0.3983	38.16	0.1753	6.18	01111
*X_1_^2^*	19.70	0.5521	1.15	0.0306	0.0457	0.9531	0.0050	0.9337	0.9176	0.1073	0.2745	0.8019	10.34	0.4582	0.4185	0.6497
*X_2_^2^*	13.67	0.6190	2.61	0.0048	62.55	0.0586	0.3174	0.5156	0.8246	0.1235	3.34	0.3933	2.55	0.4585	20.57	0.0277
*X_3_^2^*	72.78	0.2690	0.7346	0.0677	47.37	0.0903	17.75	0.0013	2.90	0.0134	0.0080	0.9657	0.1209	0.8693	40.95	0.0058
*Residual*	353.60		1.10		85.97		4.74		1.88		28.30		29.05		18.76	
*Pure error*	224.50		0.3794		25.00		0.9190		0.3559		0.4409		0.3411		13.76	
*Total*	1463.19		10.02		860.69		36.19		19.15		38.06		41.15		229.40	
*R^2^*	0.7583		0.8899		0.9001		0.8690		0.9017		0.2566		0.2939		0.9182	
*R^2^_adj_*	0.4476		0.7489		0.7717		0.7006		0.7752		0.6993		0.6138		0.8131	
C.V.*	23.95		28.28		24.75		2.08		18.96		11.52		11.47		0.3714	
A.P*	6.83		7.26		7.95		7.78		10.39		1.66		1.96		10.82	

*Note*: X_1_: puffing temperature (°C); *X*_2_, puffing time (min); *X*_3_, system temperature (°C).

Abbreviations: A.P*, adequate precision; BI, browning index; DM, dry matter; FRAP, ferric reducing antioxidant potential; GAE, gallic acid equivalent; SS, sum of square; TEAC, Trolox equivalent antioxidant capacity; TPC, total phenolic content.

Table 7 shows that higher puffing temperatures (e.g., 110°C) and longer puffing times (e.g., 15 min) generally increased the ER and decreased bulk density, reflecting the formation of light, porous, and crispy puff‐dried grapes. These observations align with the principles outlined by Ratti (2001), who emphasized that increased thermal energy elevates internal vapor pressure, promoting cell expansion during puffing. Similar trends have been reported in studies on puff‐dried fruits, where higher temperatures improved expansion but occasionally induced localized structural collapse if excessive (Chen et al. 2017; Zielinska et al. 2018). However, excessive heat exposure also led to structural degradation, as indicated by higher hardness values in certain trials. Thus, balancing puffing temperature and time is critical to achieving optimal expansion without compromising textural integrity. Antioxidant activity, evaluated by FRAP, DPPH, and ABTS assays, further demonstrated the sensitivity of bioactive compounds to puffing conditions. The highest DPPH values were recorded under moderate puffing conditions, suggesting that controlled thermal exposure favors the retention of antioxidant properties. In contrast, prolonged or excessive heating promoted oxidative degradation of phenolic compounds, consistent with earlier reports (Kumar and Karim 2019). These findings highlight the importance of carefully optimizing puffing parameters to maximize both physical quality and functional value. As expected, water activity and moisture content varied significantly with puffing temperature and time. Higher temperatures and extended durations yielded lower moisture contents, an essential factor for ensuring microbial stability and prolonging shelf life (Mujumdar 2014). The ANOVA results presented in Table 8 statistically confirm the significant effects of linear, quadratic, and interaction terms of puffing parameters on the physicochemical and textural properties of puff‐dried Sultana grapes, supporting the trends observed in experimental results.

The model exhibited strong predictive performance for several key quality parameters, particularly ER (*R* ^2^ = 0.9921), DPPH antioxidant activity (*R* ^2^ = 0.9379), and crispness (*R* ^2^ = 0.9001). In contrast, lower *R* ^2^ values for moisture content (*R* ^2^ = 0.6705) and bulk density (*R* ^2^ = 0.2935) suggest that these attributes may be influenced by additional uncontrolled factors beyond the tested variables. Puffing temperature and time exerted the most significant effects on ER, with both quadratic and interaction terms being highly influential, underscoring the importance of balancing heat input and drying duration to achieve maximum expansion. Crispness was strongly affected by system temperature and the interaction of puffing parameters, whereas prolonged puffing times reduced crispness due to overdrying. Antioxidant retention displayed a nonlinear response, with puffing and system temperatures as key determinants, highlighting the need to avoid both under‐ and overheating to minimize degradation. For BI, higher puffing temperatures and system heat intensified nonenzymatic browning reactions. These complex relationships between process parameters and product quality attributes are further illustrated by the 3D response surface plots.

The 3D response surface plots in Figure 6 illustrate the effects of puffing temperature (*X*_1_), puffing time (*X*_2_), and system temperature (*X*_3_) on key quality attributes of puff‐dried Sultana grapes, including ER, antioxidant activity (DPPH), crispness, BI, and adhesiveness. ER and crispness were strongly influenced by puffing temperature and puffing time. ER exhibited a U‐shaped response to puffing temperature. As temperature increased, ER initially decreased and reached a minimum at approximately 100°C, indicating temporary inhibition of expansion. Beyond this point, ER increased under suitable puffing conditions due to enhanced internal vapor pressure and structural expansion. The highest ER values were obtained at temperatures slightly above 100°C in combination with appropriate puffing times (8–12 min). Crispness was generally enhanced under conditions that promoted greater expansion. This trend is consistent with Ratti (2001) and Chen et al. (2017), who reported that optimal puffing temperature and duration enhance internal steam pressure, promoting cell expansion and a desirable porous structure in fruits. However, at higher temperatures (>110°C) and prolonged durations (>12 min), ER decreased due to overdrying and structural collapse, reflecting the findings of Zielińska et al. (2018) that excessive heat can lead to cell wall rupture and reduced puffing efficiency.

Antioxidant activity (DPPH) followed a nonlinear trend, with maximum retention under moderate conditions (∼100°C for 8–12 min at ∼70°C system temperature). Declines at >110°C and extended puffing times are attributed to thermal degradation of phenolic compounds. As previously emphasized by the studies of Chen et al. (2017) and Zielińska et al. (2018), controlled thermal exposure is crucial for preserving heat‐sensitive bioactive substances during swelling and drying.

The BI increased with puffing temperature and time, reaching the highest values above 105°C and 12 min, reflecting intensified Maillard reactions and caramelization. Adhesiveness decreased with higher temperature and longer time, linked to lower residual moisture, although excessive thermal stress eventually caused structural collapse and a brittle product.

On the basis of these findings, puffing temperature (*X*_1_), puffing time (*X*_2_), and system temperature (*X*_3_) were selected as independent variables for process optimization. Using a second‐order polynomial model within the tested ranges (*X*_1_: 90–110°C; *X*_2_: 5–15 min; *X*_3_: 60–80°C), optimization was performed via desirability function and superimposition approaches. The model targeted maximum ER and crispness, in‐range adhesiveness and DPPH activity, and minimal BI. The optimal conditions were identified as 104°C puffing temperature, 5 min puffing time, and 76°C system temperature, with a composite desirability of 0.814.

**FIGURE 7:**
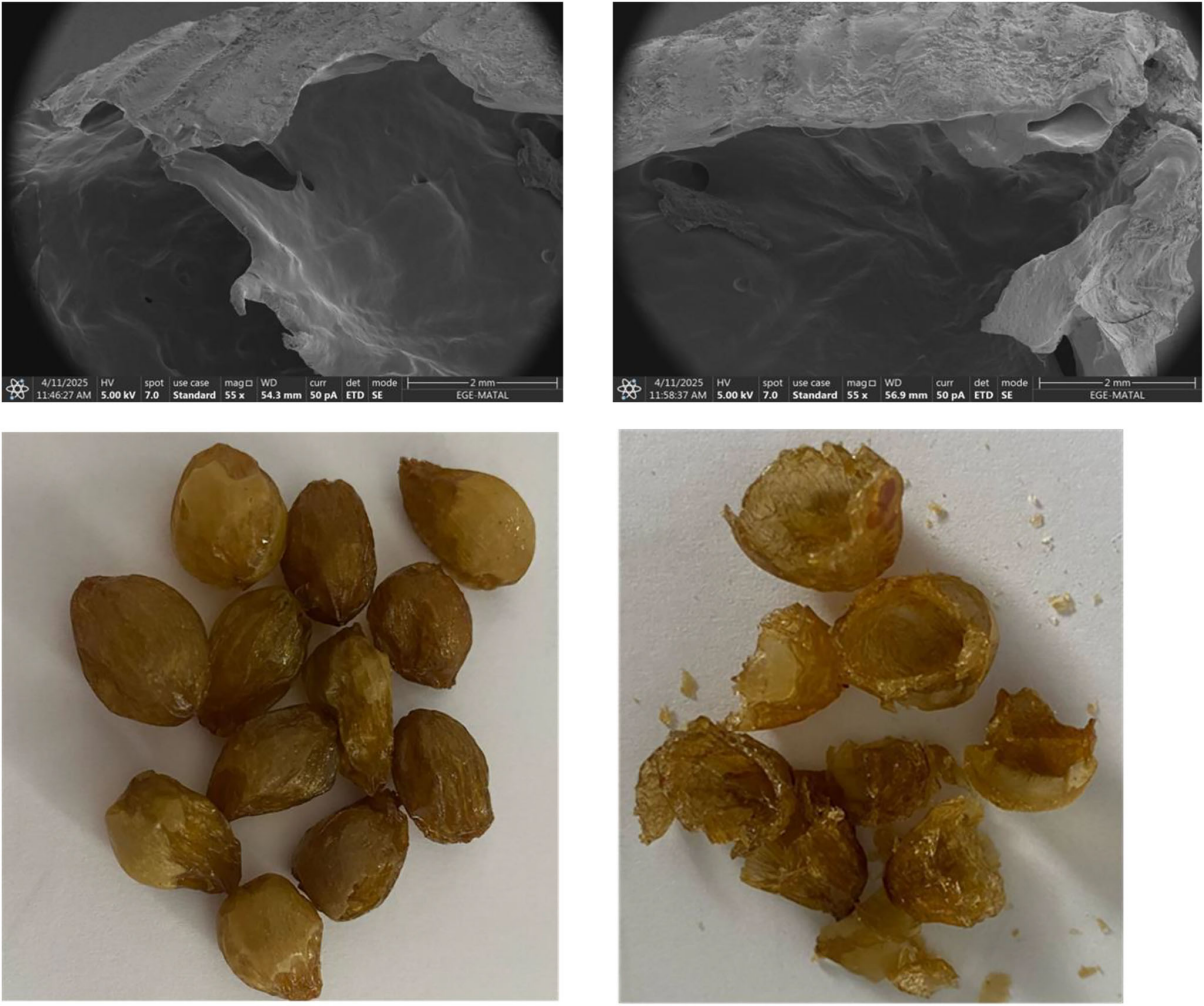
Photographs and SEM images of grapes dried under optimum conditions.

Experimental validation under these conditions confirmed the model's accuracy, as the experimentally obtained ER, crispness, adhesiveness, DPPH, and BI values from five replicates showed no significant difference from the predicted responses (*p* > 0.05). Representative internal and external product images, along with SEM micrographs of grapes processed under the optimized conditions, are presented in Figure 7.

Figure 7 presents SEM images (top row) and macroscopic photographs (bottom row) of grape samples processed under the optimized drying conditions. The SEM micrographs reveal a well‐developed porous microstructure with expanded cellular matrices and reduced compactness, demonstrating effective internal pressure build‐up and structural transformation during explosion puff drying. These features indicate successful drying, where cellular integrity was largely preserved, whereas volumetric expansion was promoted. The macroscopic images corroborate these findings, showing grape chips with a light, brittle texture and uniform puffing across the samples. The hollowed interiors and crisp outer surfaces further confirm that the optimized pre‐drying and puffing parameters enabled the formation of a desirable porous network. This structure not only enhances texture but may also improve rehydration capacity. Collectively, these results validate that the hybrid pre‐drying approach combined with explosion puff drying produced grape chips with favorable structural and visual characteristics, making them suitable as functional snack products.

## 4 Conclusion

This study systematically investigated the combined effects of hybrid pre‐drying and explosion puff drying (EPD) on the physicochemical, structural, textural, and functional properties of Sultana grape chips. The results clearly demonstrated that both the selection of the pre‐drying method and the optimization of EPD parameters play a critical role in determining final product quality.

Among the evaluated pre‐drying strategies, the hybrid approach (MD at 360 W followed by HAD at 60°C) provided the most favorable balance between rapid moisture removal and controlled thermal exposure. This combination effectively preserved phenolic compounds and antioxidant activity while promoting desirable structural characteristics, including lower bulk density, higher ER, and improved crispness.

Optimization of the EPD process further revealed that puffing temperature and time were the dominant factors governing structural expansion, texture development, and browning behavior. The identified optimum conditions (104°C puffing temperature, 5 min puffing time, and 76°C system temperature) enabled the production of grape chips with a well‐developed porous structure, high crispness, reduced adhesiveness, and minimal nonenzymatic browning, while maintaining significant antioxidant capacity.

The improved product quality and structural properties obtained with hybrid drying were also supported by SEM observations, which confirmed the formation of a more homogeneous and expanded microstructure. In addition, the hybrid drying approach demonstrated favorable energy performance based on SMER values. This study provides a scientifically grounded and experimentally validated framework for the production of high‐quality, functional fruit snacks using hybrid drying and EPD technologies. The findings contribute to a better understanding of heat–mass transfer‐driven structural transformations in high‐sugar fruits and offer practical guidance for industrial‐scale applications aiming to improve product quality, process efficiency, and sustainability.

## Author Contributions

**Özgül Altay**: conceptualization, formal analysis, validation, investigation, methodology, writing and editing original draft. **Figen Kaymak‐Ertekin**: funding acquisition, project administration, supervision, writing and editing original draft.

## Funding

This study was supported by the Scientific and Technological Research Council of Türkiye (Grant number TÜBİTAK 124O716). The authors gratefully acknowledge the support of the Council of Higher Education (YÖK), Türkiye, through the 100/2000 Doctoral Scholarship Program.

## Ethics Statements

Ethical approval for this study was obtained from the Ege University Natural and Applied Sciences Ethics Committee (Decision No.: 2874, Date: 28/04/2025).

## Conflicts of Interest

The authors declare no conflicts of interest.
